# Intracranial aneurysm’s association with genetic variants, transcription abnormality, and methylation changes in *ADAMTS* genes

**DOI:** 10.7717/peerj.8596

**Published:** 2020-02-14

**Authors:** Shi Chen, Mengqi Li, Wenqiang Xin, Shengze Liu, Linfei Zheng, Yan Li, Mengyao Li, Mengxiong Zhan, Xinyu Yang

**Affiliations:** 1 Department of Neurosurgery, Tianjin Medical University General Hospital, Tianjin, China; 2 Department of Neurosurgery, Fuzhou Second Hospital Affiliated to Xiamen University, Fuzhou, Fujian, China; 3 Key Laboratory of Post-Neurotrauma Neurorepair and Regeneration in Central Nervous System, Ministry of Education of China, Tianjin, China; 4 Tianjin Neurological Institute, Tianjin, China; 5 Fuzhou Medical Center of Neuroscience, Fuzhou, China; 6 Department of Radiology, Zhenning People’s Hospital, Zhengning, Gansu, China; 7 Department of Neurology, Wuzhong People’s Hospital, Wuzhong, Ningxia, China

**Keywords:** ADMTSL1, Bioinformatics, Hemorrhagic stroke, Single-nucleotide variants

## Abstract

**Purpose:**

The development of intracranial aneurysm (IA) has been linked to genetic factors. The current study examines the potential role of genes encoding disintegrin and metalloproteinase using thrombospondin motifs (ADAMTS) in IA development.

**Material and Methods:**

High-throughput whole-genome and whole-exome sequencing were used when screening for deleterious single-nucleotide variants (SNVs) in *ADAMTS* genes using samples from 20 Han Chinese patients: 19 with familial IA and one patient with sporadic IA. The variant frequencies in these subjects were compared to those in control individuals found in the Genome Aggregation Database. Transcriptome sequencing and methylation sequencing data were retrieved from the Gene Expression Omnibus (GEO) database to identify differentially expressed *ADAMTS* genes and their methylation sites. We predicted the network of interactions among proteins encoded by the overlapping set of *ADAMTS* genes showing deleterious variants and both differential expression and abnormal methylation in IA. Possible candidate proteins linked to IA were validated using Western blot analysis. The associations between IA and SNVs rs11750568 in *ADAMTS2*, as well as rs2301612 and rs2285489 in *ADAMTS13*, were verified using the Sequenom MassArray system on a separate sample set of 595 Han Chinese patients with sporadic IA and 600 control individuals.

**Results:**

A total of 16 deleterious variants in 13 *ADAMTS* genes were identified in our patients, and seven of these genes overlapped with the genes found to be differentially expressed and differentially methylated in the GEO database. Protein–protein interaction analysis predicted that ADAMTSL1 was at the center of the seven genes. ADAMTSL1 protein was lower expressed in IA tissue than in the control cerebral artery. Frequencies of the IA-related SNVs rs11750568 in ADAMTS2 and rs2301612 and rs2285489 in *ADAMTS13* were not significantly different between sporadic IA patients and controls.

**Conclusion:**

IA is associated with genetic variants, differential expression, and abnormal methylation in *ADAMTS* genes, *ADAMTSL1* in particular.

## Introduction

Intracranial aneurysm (IA) is a major cause of hemorrhagic stroke. Acute cerebrovascular IA events pose a notable threat to younger individuals, leading to a decrease in productive years and a tremendous burden on society (Johnston, Selvin & Gress, 1998; Korja & Kaprio, 2016; Lawton & Vates, 2017; Rivero-Arias, Gray & Wolstenholme, 2010). Systematic reviews and meta-analyses indicate that the overall worldwide prevalence of unruptured IA is 3.2% (95% CI [1.9–5.2]) (Vlak et al., 2011). Individuals with a family history of aneurysmal subarachnoid hemorrhage are at a higher risk of unruptured IA and aneurysmal subarachnoid hemorrhage (Bor et al., 2008; Bor et al., 2014; Vlak et al., 2011), indicating that genetics may play a contributing factor in IA. To date, the underlying mechanisms by which genetic factors contribute to IA remain poorly understood (Nakaoka et al., 2014).

A previous genome-wide association study identified a network of disintegrin and metalloproteinase with thrombospondin motifs (ADAMTS) associated with IA (Arning et al., 2012). Members of the ADAMTS protein family have multiple biological functions related to the structure and remodeling of the brain’s arterial wall, including extracellular matrix degradation (Binder et al., 2017), modulation of endothelial cell angiogenesis (Tang et al., 2017), vascular smooth muscle cell migration (Wang et al., 2009), and inflammation (Lemarchant et al., 2016; Zhang, Lin & Wei, 2015). ADAMTS-like genes (ADAMTSLs) produce proteins similar to those encoded by ADAMTS genes, only without catalytic domains, and may be responsible for regulating activities of ADAMTS (Apte, 2009; Dubail & Apte, 2015). ADAMTSL1 was first identified in 1997 and was called punctin (Blobel, 1997; Hirohata et al., 2002). ADAMTSL1 is distinct from other genes in the ADAMTS family, and binds to the extracellular matrix in a spatially-specific manner (Hirohata et al., 2002). Because IA development is caused by changes such as the degeneration of the extracellular matrix (Sawyer et al., 2016), it is possible that ADAMTS protein dysfunction may contribute to the development of IA, but the exact mechanisms are unclear.

In the current study, we aimed to determine the relationship between *ADAMTS* genes and IA using multiple approaches, including sequence polymorphism, expression, and methylation of genes. Our results provided several testable hypotheses to guide future research.

## Material and Methods

### Ethics statement

All experimental protocols were in compliance with the Declaration of Helsinki and were approved by the Institutional Review Boards and Ethics Committees of Tianjin Medical University General Hospital (IRB2019-KY-134) and Fuzhou Second Hospital Affiliated to Xiamen University (SQ2018-004). All subjects or their legal guardians gave written informed consent.

### Determination of deleterious variants in ADAMTS genes

**Figure 1:**
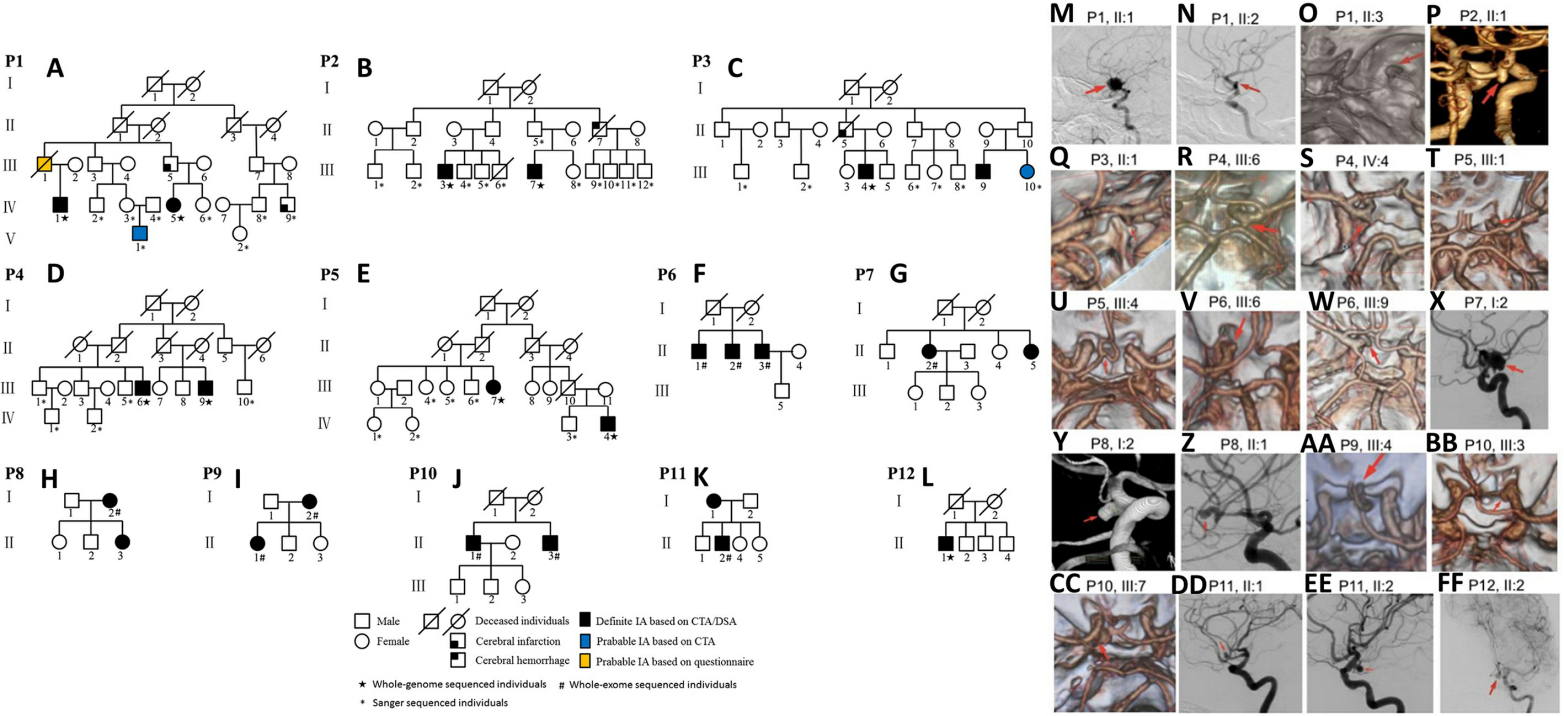
Discovery corhort and imaging diagnosis. (A–L) Diagrams showing 20 cases of intracranial aneurysm (IA), collected from 11 pedigrees. (M-FF) Imaging diagnosis: Digital subtraction angiography (DSA) or computed tomography angiography (CTA) images of 20 Han Chinese IA patients in this study.

This study included 20 Han Chinese IA patients from 11 families with two or more affected members in each family. The cerebral aneurysm was confirmed using digital subtraction angiography or computed tomography angiography. Whole-genome sequencing (WGS) and whole-exome sequencing (WES) were both conducted in 10 patients, respectively (Fig. 1, Data S1). Sequencing data that met quality criteria were analyzed for deleterious variants. The potential harmfulness of variants within exonic or splicing regions was predicted using SIFT (Vaser et al., 2016), Polyphen (Adzhubei, Jordan & Sunyaev, 2013), MutationTaster (Schwarz et al., 2010) and CADD (Rentzsch et al., 2018). Variants were considered deleterious if they were deemed harmful by any of the algorithms. *ADAMTS* genes were screened for deleterious variants using data from non-IA controls in the Genome Aggregation Database (gnomAD, http://gnomad.broadinstitute.org/) (Zlotogora, Patrinos & Meiner, 2018). Fisher‘s exact test was used to determine significant differences in the frequencies of deleterious *ADAMTS* variants between our IA patients and the gnomAD control data.

### Identification of differentially expressed ADAMTS genes using microarray data from the GEO database

We collected IA tissue mRNA expression data from 15 patients and control cerebral artery tissues from 15 individuals in the GEO database (http://www.ncbi.nlm.nih.gov/geo/GSE75436; Table S1). Differences in mRNA levels between the two groups were compared using the online analysis tool GEO 2R (http://www.ncbi.nlm.nih.gov/geo/geo2r/?acc=GSE75436) (Davis & Meltzer, 2007). Differentially expressed genes were defined as *P* < 0.05 and —logFC— ≥0.5, where FC is the fold change in gene expression level. All differentially expressed genes in the *ADAMTS* family were screened and presented in a heat map.

### Differential methylation of ADAMTS genes in IA and cerebral artery tissues

High-throughput methylation data from IA and cerebral artery tissues were obtained from the GEO database (GSE75434) (Yu et al., 2017). This set of data included nine IA tissues and nine matched cerebral artery tissues from different patients. GEO 2R was used to identify differentially methylated sites between IA and cerebral artery tissues. *P* < 0.05 was regarded as statistically significant.

### Predicting ADAMTS protein-protein interactions in IA

We used the Search Tool for the Retrieval of Interacting Genes/Proteins (STRING, version 11.0; https://string-db.org/) to predict protein interactions. We analyzed the subset of ADAMTS genes that contained deleterious variants, that were differentially expressed, and were methylated in IA.

### Analysis of ADAMTSL1 expression in IA and cerebral artery tissues

IA specimens were donated by three patients who underwent clipping of IA at Fuzhou Second Hospital Affiliated to Xiamen University. Control cerebral artery samples were taken from autopsies performed in the Department of Pathology at Fuzhou Second Hospital Affiliated to Xiamen University. All specimen donors provided written informed consent. The study protocol was approved by the hospital’s Ethics Committee (SQ2018-004).

Tissue samples were homogenized and 20 µl was fractionated using SDS-PAGE (5% stacking gel, 10% separating gel). Broad-range pre-stained protein markers were also separated, and electrophoresis was conducted for 1.5–2 h in a buffer containing Tris-aminomethane, glycine, and 0.1% SDS. After electrophoresis, proteins were transferred onto polyvinylidene difluoride membranes as previously described. Block Ace (orb436742; Biorbyt, Cambridge, UK) was included to determine non-specific binding. The transferred membranes were incubated overnight at 4 °C with a primary rabbit antibody against human ADAMTSL1 (1:1,000; ab155597, Abcam). The membranes were washed extensively, then incubated for 1 h at room temperature with a universal biotinylated streptavidin-HRP secondary antibody against rabbit IgG (ab97051, Abcam). Bands were detected and quantitated using the ChemiDoc™ MP System (170-8280, Bio-Rad, USA).

### Determining differing ADAMTS SNVs between sporadic IA patients and control subjects in a Han Chinese population

Clinical information and blood samples from 595 IA patients were obtained from CMAD (http://database.cmadtj.com/). IA was confirmed using subtraction or computed tomography angiography. A total of 600 control subjects were selected from a database in the Physical Examination Center of Tianjin Medical University General Hospital. The control subjects’ medical records were checked to ensure that they had no indication of IA.

Genomic DNA was extracted from the blood samples and stored at −80 °C. The SNV rs11750568 in *ADAMTS2* and the SNVs rs2301612 and rs2285489 in *ADAMTS13*, previously reported (Arning et al., 2016), were genotyped using the Sequenom MassArray system (BioMiao Biological Technology, Beijing, China). Data were analyzed using MassArray Typer 4.0 (Agena Bioscience, San Diego, USA). The association between *ADAMTS2* and *ADAMTS13* variants and IA was examined using Plink 2.0 (Supplemental Information) (Purcell et al., 2007).

### Statistical analysis

Differences in mRNA levels and methylation sites were compared between the two groups using GEO 2R, and *P* < 0.05 was considered potentially differentially expressed or methylated. The Benjamini & Hochberg multiple correction method (false discovery rate) was used (Madar & Batista, 2016). Protein levels, determined by Western blot, were compared using Student’s *t*-test in SPSS 22.0 (64-bit edition; IBM, Chicago, IL, USA). Chi-squared test and multiple-test correction for analyses of rs11750568, rs2301612, and rs2285489 were performed in Plink 2.0. *P* < 0.05 was regarded as statistically significant.

## Results

### Deleterious variants in the ADAMTS gene family

A total of 16 variants in the *ADAMTS* family were found in exons and other functional areas (Table 1). Further comparisons against gnomAD control data showed that these deleterious variants were enriched in the IA patients from 11 families in the present study (Table 2).

**Table 1:** Prediction of single-nucleotide variations (SNVs) related to intracranial aneurysm.

**ID**	**REF**	**ALT**	**Amino acid change**	**Gene**	**Function**	**Prediction tool**			
						**SIFT**^a^	**Mutation Taster**^b^	**gerp++gt2**^c^	**CADD**^d^
rs746852468	G	A	Ser >Leu	ADAMTS4	exonic	0.144,T	0.994,D	4.34	14.88
rs61753558	A	T	Leu >Pro	ADAMTS12	exonic	0.0,D	1.000,D	5.57	22.0
rs147540204	G	C	Pro >Ala	ADAMTS6	exonic	0.059,T	1,D	4.86	16.94
rs368690576	C	T	Gly >Ser	ADAMTS2	exonic	0.107,T	0.951,D	4.72	22.9
rs2271211	C	T	Val >Met	ADAMTS2	exonic	0.087,T	0.989,N	4.23	20.6
rs141581125	G	A	Asp >Asn	ADAMTSL1	exonic	0.479,T	0.978,D	5.77	18.75
rs74797959	C	T	Arg >Trp	ADAMTS14	exonic	0.103,T	0.895,D	.	15.30
rs150906283	A	T	N/A	ADAMTS8	splicing	.	.	.	11.19
rs185269810	G	C	Glu >Gln	ADAMTS15	exonic	0.02,D	1.000,D	3.49	20.5
rs372136438	G	A	Arg >Ter	ADAMTS20	exonic	.	1,A	2.86	40
rs186123571	G	A	Thr >Ile	ADAMTS7	exonic	0.098,T	0.968,D	4.57	14.50
rs2127898	G	A	Thr >Met	ADAMTS7	exonic	0.005,D	0.031,P	2.85	16.30
rs77028575	G	A	Arg >His	ADAMTSL3	exonic	0.005,D	1.000,D	5.45	25.1
rs544641967	C	G	Gly >Arg	ADAMTS18	exonic	0.0,D	1.000,D	5.54	28.1
rs540472609	C	T	Cys >Tyr	ADAMTS18	exonic	0.003,D	0.815,D	4.23	14.96
rs200029215	G	A	Pro >Leu	ADAMTSL5	exonic	0.0,D	1,D	4.28	19.09

**Notes.**

Abbreviations ALT alternative allele REF reference

a SIFT score indicates whether the variation is likely to cause changes in protein structure or function: “D”, deleterious (sift ≤ 0.05); “T”, tolerated (sift > 0.05).

b MutationTaster predicts the effect of the mutation on the protein sequence: “A”, “disease_causing_automatic”; “D”, “disease_causing”; “N”, “polymorphism”; “P”, ”polymorphism_automatic”.

c Variations with a gerp++gt2 score > 2 are considered conservative.

d CADD score >15 means that the variation affects protein function.

**Table 2:** Deleterious single-nucleotide polymorphisms in ADAMTS genes in patients with intracranial aneurysm.

**SNP_ID**	**Gene**	**Polymorphic locus**	**Our study**	**GnomAD**	**P (Fisher’s exact test)**	**OR**	**95% CI**	
			**risk allele/ normal allele**	**risk allele/ normal allele**			**Lower**	**Upper**
rs746852468	ADAMTS4	A	A/G=1/19	T/C=7/244892	0.001	1,841	216	15,697
rs61753558	ADAMTS12	T	T/A=1/19	T/A=548/245962	0.044	24	3	177
rs147540204	ADAMTS6	C	C/G=1/19	C/G=318/245638	0.026	41	5	305
rs368690576	ADAMTS2	T	T/C=1/19	T/C=6/149126	0.001	1,308	150	11,391
rs2271211	ADAMTS2	T	T/C=1/20	T/C=409/234580	0.034	30	4	226
rs141581125	ADAMTSL1	A	A/G=1/20	A/G=85/245886	0.007	152	20	1,150
rs74797959	ADAMTS14	T	T/C=3/17	T/C=1077/246088	<0.001	40	11	138
rs150906283	ADAMTS8	T	T/A=3/17	T/A=628/245972	<0.001	69	20	236
rs185269810	ADAMTS15	C	C/G=1/19	C/G=692/235086	0.057	18	2	134
rs372136438	ADAMTS20	A	A/G=1/19	A/G=14/220938	0.001	831	104	6,635
rs186123571	ADAMTS7	A	A/G=1/19	no data				
rs2127898	ADAMTS7	A	A/G=12/8	A/G=80464/245590	0.12	2	0.8	4.9
rs77028575	ADAMTSL3	A	A/G=3/17	A/G=417/246136	<0.001	104	30	357
rs544641967	ADAMTS18	G	G/C=1/19	G/C=4/245924	<0.001	3,236	346	30,303
rs540472609	ADAMTS18	T	T/C=1/19	T/C=6/171256	0.001	1,502	173	13,081
rs200029215	ADAMTSL5	A	A/G=1/19	A/G=103/213450	0.01	109	14	822

**Notes.**

Abbreviations CI confidence interval GnomAD genome aggregation database OR odds ratio SNP_ID single-nucleotide polymorphism identification

### ADAMTS genes differentially expressed between IA patients and controls

**Figure 2:**
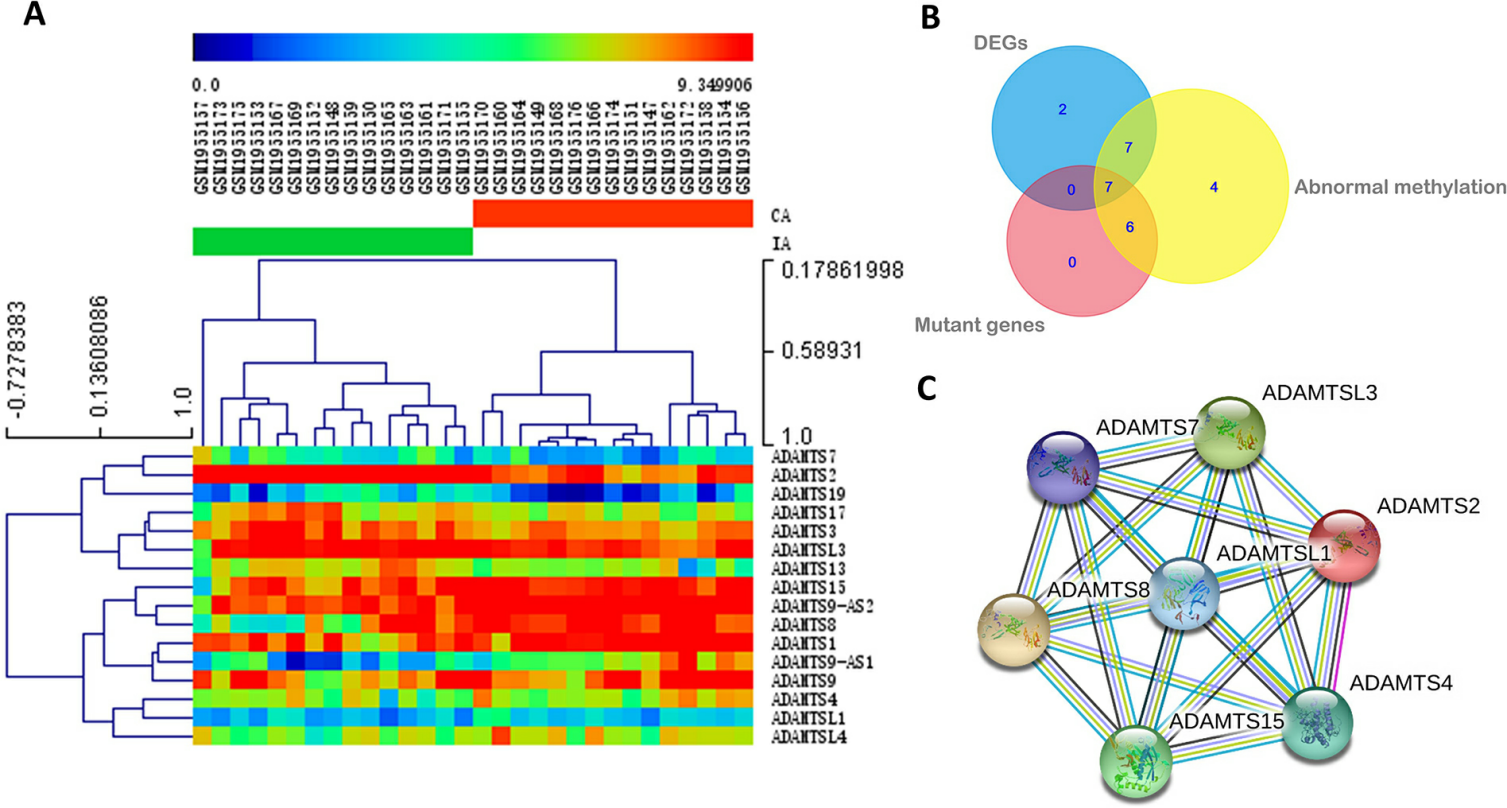
Differentially expressed ADAMTS genes and the network of interactions among proteins encoded by the overlapping genes. (A) Heatmap of differentially expressed ADAMTS genes. Different colors represent different levels of expression. The row above the heatmap corresponds to sample numbers, and the column on the right side of the heatmap corresponds to differentially expressed genes. CA, cerebral artery; IA, intracranial aneurysm. (B) Overlap among ADAMTS genes with deleterious variants (mutant genes), ADAMTS genes differentially expressed (DEGs) in IA, and ADAMTS genes abnormally methylated in IA. Seven genes overlapped among the three sets: ADAMTS15, ADAMTS2, ADAMTS4, ADAMTS7, ADAMTS8, ADAMTSL1, and ADAMTSL3. (C) A network of interactions among proteins encoded by the seven overlapping genes was predicted using String (https://string-db.org/) in order to identify the genes most likely to be relevant to IA. ADAMTSL1 is at the center of this network, suggesting that it acts upstream of the other overlapping genes.

The online analysis tool GEO 2R was used to analyze data collected from GEO. Among the 16 potentially differentially expressed *ADAMTS* genes, *ADAMTS9-AS1, ADAMTS8, ADAMTS9-AS2, ADAMTS1, ADAMTS9, ADAMTS15, ADAMTS4, ADAMTSL4,* and *ADAMTSL1* were upregulated in IA; and *ADAMTS3, ADAMTS17*, *ADAMTS13, ADAMTSL3, ADAMTS7, ADAMTS19,* and *ADAMTS2* were downregulated. The changes remained significant after multiple testing corrections for *ADAMTS9-AS1, ADAMTS8, ADAMTS9-AS2, ADAMTS1, ADAMTS13, ADAMTS7, ADAMTS19*, *and ADAMTS2* (Table 3). Differentially expressed *ADAMTS* genes are shown in Table 3 and Fig. 2A.

**Table 3:** ADAMTS genes differentially expressed between individuals with intracranial aneurysm and controls.

**Gene symbol**	**Adjusted *P* value**	***p***	***t***	**log FC**
**ADAMTS9-AS1**	2.88 × 10^−4^	<0.001	5.433435	3.296323
**ADAMTS8**	2.63 × 10^−3^	<0.001	4.393505	2.777007
**ADAMTS9-AS2**	1.58 × 10^−5^	<0.001	6.82637	1.786802
**ADAMTS1**	5.44 × 10^−3^	<0.001	4.049965	1.650133
ADAMTS9	1.38 × 10^−1^	0.026	2.330043	1.404467
ADAMTS15	1.21 × 10^−1^	0.022	2.411667	1.096499
ADAMTS4	2.04 × 10^−1^	0.047	2.063526	0.751622
ADAMTSL4	1.58 × 10^−1^	0.032	2.241619	0.708539
ADAMTSL1	1.42 × 10^−1^	0.028	−2.311788	−0.678263
ADAMTS3	1.05 × 10^−1^	0.018	−2.49951	−0.76742
ADAMTS17	7.83 × 10^−2^	0.012	−2.67964	−0.77946
**ADAMTS13**	2.52 × 10^−2^	0.002	−3.29649	−1.24966
ADAMTSL3	5.51 × 10^−2^	0.007	−2.87834	−1.36329
**ADAMTS7**	3.74 × 10^−2^	0.004	−3.09323	−1.44272
**ADAMTS19**	2.39 × 10^−2^	0.002	−3.32586	−1.77612
**ADAMTS2**	8.23 × 10^−9^	2.86 × 10^−12^	−11.0591	−2.87598

**Notes.**

Abbreviations log FC log fold change t statistic from Student’s t test

The data sourced from GEO database (http://www.ncbi.nlm.nih.gov/geo/GSE75436). Differentially expressed genes identified with multiple correction are shown in bold.

### Sites in ADAMTS genes differentially methylated between IA and cerebral artery tissues

A comparison between the IA samples and the cerebral artery revealed a total of 299 potentially differentially methylated sites in 24 ADAMTS genes. Among these sites, 193 were upregulated and 106 were downregulated (Table S2).

### The subset of ADAMTS genes with deleterious variants, differential expression, and differential methylation in IA

Seven *ADAMTS* genes were found to contain deleterious genetic variants and were differentially expressed and methylated in IA: *ADAMTS15, ADAMTS2, ADAMTS4, ADAMTS7, ADAMTS8, ADAMTSL1,* and *ADAMTSL3* (Fig. 2B). Protein-protein interaction prediction suggested that *ADAMTSL1* was at the center of the network of all seven genes (Fig. 2C).

### Lower expression of ADAMTSL1 in IA issue

**Figure 3:**
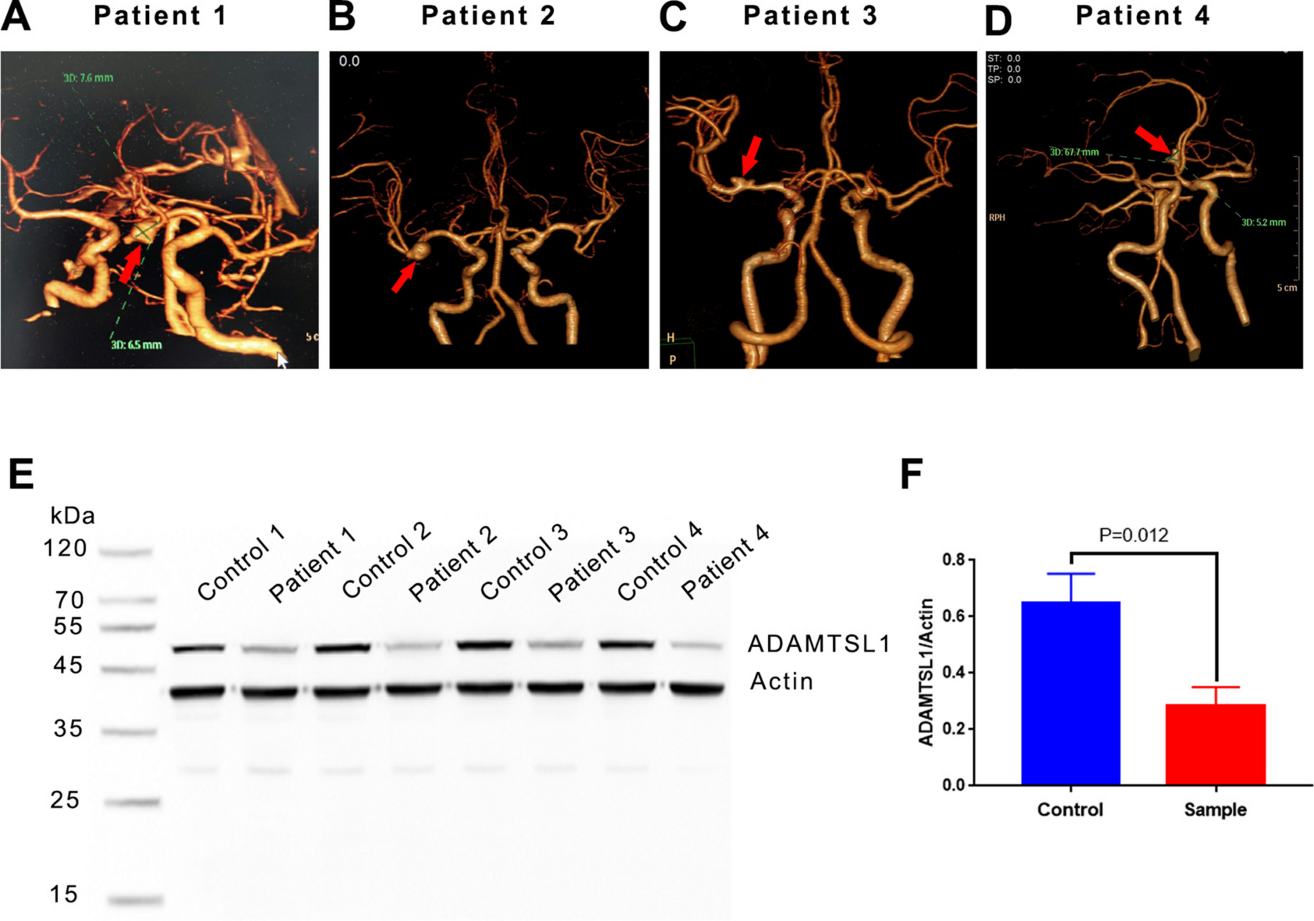
Expression of ADAMTSL1 in IA issue and cerebral artery. (A) Identification of IA tissue based on computed tomography angiography: Patient 1, Patient 2, Patient 3 and Patient 4. Red arrows indicate the location of IA. (B) ADAMTSL1 was expressed at lower levels in IA tissue than in cerebral artery.

Based on the prediction that ADAMTSL1 plays a key role in ADAMTS genes in IA, we investigated its expression in IA and cerebral artery tissue (Fig. 3). The results showed lower ADAMTSL1 levels in IA tissue than in a cerebral artery.

### Determining specific SNPs in sporadic IA patients and control individuals from a Han Chinese population

The following ADAMTS gene variants were not associated with IA in a cohort of Han Chinese patients and controls even after multiple corrections (Table 4): allele A in rs11750568, OR 0.9696 (95% CI [0.7464–1.26], *P* = 0.82); allele T in rs2285489, OR 0.93 (95% [0.7309–1.187], *P* = 0.56); and allele G in rs2301612, OR 0.96 (95% [0.77–1.20], *P* = 0.71).

**Table 4:** SNVs rs11750568 in ADAMSTS2 and rs2301612 and rs2285489 in ADAMSTS13.

**SNP**	**A1**	**TEST**	**OR**	**SE**	**L95**	**U95**	***P***	**BONF**	**FDR_BY**^a^
rs11750568	A	ADD	0.970	0.133	0.746	1.26	0.817	1	1
rs2285489	T	ADD	0.931	0.124	0.731	1.187	0.565	1	1
rs2301612	G	ADD	0.958	0.15	0.765	1.2	0.710	1	1

**Notes.**

a Multiple-testing correction methods were BONF and FDR_BY.

Abbreviations SNVs single- nucleotide variants BONF Bonferroni single-step adjusted *P*-values FDR_BY Benjamini & Yekutieli (2001) step-up FDR control

## Discussion

A total of 16 deleterious variants in 13 *ADAMTS* genes were found to be associated with IA (Table 2). Sixteen potentially differentially expressed genes were discovered from transcriptomics data and 299 potentially differentially methylated sites in 24 *ADAMTS* genes were identified from methylation sequencing data taken from IA and cerebral artery samples in the GEO database. An overlapping set of seven *ADAMTS* genes were found to contain deleterious variants and were differentially expressed and methylated in IA: *ADAMTS15, ADAMTS2, ADAMTS4, ADAMTS7, ADAMTS8, ADAMTSL1,* and *ADAMTSL3*. A genome-wide association study and other molecular biology studies have recognized *ADAMTS15* as a candidate gene for IA in a Japanese population (Yan et al., 2015). Surprisingly, this gene did not show a significant association with IA in our cohort (*P* = 0.057; Table 2). This negative result is likely due to a lack of statistical power, and should be verified in larger cohorts. The *ADAMTS2* variant rs11750568 has been previously associated with IA and pediatric stroke (Arning et al., 2012; Arning et al., 2016). ADAMTS4 protein and mRNA are expressed at higher levels in thoracic aortic aneurysm and dissection tissues than in control aortic tissues, and increased ADAMTS4 levels can degrade versican and facilitate macrophage invasion (Ren et al., 2013). Human aortic aneurysm induction is related to upregulated ADAMTS-7 and downregulated COMP in the ADAMTS7/COMP pathway. In patients with peripheral arterial occlusion, levels of ADAMTS8 and macrophages in the blood are lower if an aortic aneurysm is present (Lamblin et al., 2010). *ADAMTSL3* is a candidate gene for diabetes, which is, in turn, a risk factor for IA (Jambaljav et al., 2018; Lindgren et al., 2013).

A previous study of *ADAMTS* gene polymorphisms and IA risk in a European population identified three risk alleles: allele A at rs11750568 in *ADAMTS2* (OR 1.32, *P* = 0.006), allele T at rs2301612 (OR 1.26, *P* = 0.011), and allele G at rs2285489 in *ADAMTS13* (OR 1.24, *P* = 0.02) (Arning et al., 2016). In our present study of a Han Chinese population, there were no significant differences between the risk alleles of the IA patients and controls after multiple testing corrections (Table 4). One possible explanation for this is that risk alleles for IA may differ among different populations. It is also possible that the current or previous studies’ designs were underpowered.

The *ADAMTSL* gene *ADAMTSL1* was expressed at lower levels in IA tissue than in the cerebral artery in both our patients and in data from GEO (Table 3). ADAMTSL1 is well-positioned to have a substantial influence on IA development since it is predicted to be at the center of the protein-protein network (Fig. 2C). The resemblance of ADAMTSL proteins to ADAMTS proteases and their matrix binding properties indicate a potential function in ADAMTS regulation (Apte, 2009). Therefore, we speculate that ADAMTSL proteins, including ADAMTSL 1 through 6 and papilin, may act as upstream regulators of ADAMTS proteins (Apte, 2009; Dubail & Apte, 2015; Kelwick et al., 2015). Additional experiments are needed for further verification. ADAMTSL1’s binding to the extracellular matrix (Hirohata et al., 2002) may influence the degradation of extracellular matrix levels, which may contribute to IA development (Sawyer et al., 2016). Lower levels of *ADAMTSL1* mRNA and protein in IA tissue may be associated with differential methylation (Yong, Hsu & Chen, 2016). Our results suggest that *ADAMTSL1* may regulate the influence of *ADAMTS* genes in IA. However, this speculation must be tested directly in future studies.

There are some limitations to this study. First, although we present evidence that *ADAMTS* are novel candidate genes associated with IA, these findings should be verified and explained using additional mechanistic studies. Second, we did not conduct experiments to directly verify whether *ADAMTSL1* influences the levels or activities of *ADAMTs* genes. Third, the associations between IA and *ADAMTS* variants should be explored in larger and more ethnically diverse samples.

## Conclusion

IA development is associated with genetic variants, differential expression, and abnormal methylation of ADAMTS genes, specifically ADAMTSL1.

##  Supplemental Information

10.7717/peerj.8596/supp-1 Data S1 Supplemental data

10.7717/peerj.8596/supp-2 Supplemental Information 1 Original image of western-blotting

10.7717/peerj.8596/supp-3 Table S1 Background information of microarray data obtained from Gene Expression Omnibus

10.7717/peerj.8596/supp-4 Table S2 Differentially methylated sites in the ADAMTS gene family

## Additional Information and Declarations

### Data Availability

The following information was supplied regarding data availability: Data is available at NCBI GEO: GSE75436, GSE75434.
