# Supplementary material for: Intracranial aneurysm’s association with genetic variants, transcription abnormality, and methylation changes in ADAMTS genes
Source: PeerJ. 2020 Feb 14;8:e8596. doi: 10.7717/peerj.8596 (PMC7025701; doi:10.7717/peerj.8596)
Supplement: Data S1 [file peerj-08-8596-s001.docx]

**1. PLINK commands**

plink --file 1 --out 1.maf –freq(Minimum allele statistics)

plink --file 1 --out 1.hw –hardy(Hardy-Weinberg equilibrium)

plink --file 1 --out 1.assoc --assoc --ci 0.95 --adjust --hwe 0.001(Chi-square test)

plink --file 1 --out 1.logistic --logistic --ci 0.95 –adjust(Allele gene logistic regression)

plink --file 1 --out 1.logistic_covar --logistic --ci 0.95 --adjust --covar covar(Allele gene logistic regression)

plink--file 1--out 1.model --model --cell 1--ci 0.95(Genotype statistics)

plink--file 1--out 1.fisher--fisher--ci 0.95--adjust --hwe 0.001(Fisher test)

**2. Data generation and processing**

2.1 Whole genome sequencing and whole exome sequencing

3.1.1 Whole genome sequencing

3.1.2 Whole exome sequencing

2.2 Quality control checks on raw sequence data

The quality of raw reads from high throughput sequencing pipelines were checked using the FastQC Version 0.11.4:

fastqc –f fastq $fq_file1 $fq_file2 –o out_dir

All of the raw data of the samples passed the criteria of the “good sequence data”.

2.3 Whole genome and exome alignment and BAM processing

Data was aligned with bwa v0.7.13 to the GRCh37 (hg19) reference genome. The reference fasta file was first indexed:

bwa index –a bwtsw $ref_fa

Then, for each fastq file, a suffix-array index (sai) file was created

bwa aln –q 15 –f $sai_file $ref_fa $fq_file

Aligned SAM files4 were created using ‘bwa sampe’ for paired-end reads. The maximum insert size was set to be three times the expected insert size.

bwa sampe –a $max_insert_size –f $sam $ref_fa $sai_files $fq_files

Raw SAM files was converted to BAM, name-sorted, mate information fixed, coordinate sorted:

samtools view –bS $sam | \

samtools sort –n –o – samtools_nsort_tmp | \

samtools fixmate /dev/stdin /dev/stdout | \

samtools sort - > $bam

The run-level alignment BAMs are improved in various ways to help increase the quality and speed of subsequent SNP calling that will be carried out on them. Duplicated molecules in the BAM files were located and flag with Picard v1.93 (http://broadinstitute.github.io/picard/).

java $jvm_args –jar picard-tools-1.93/MarkDuplicates.jar \

I=$bam O=$dedup.bam M=$bam.marked.mertics \

REMOVE_DUPLICATES=true ASSUME_SORTED=true \

TEMP_DIR=dedup_tmp

Then, reads are locally rerealigned around known indels using GATK IndelRealigner.

java $jvm_args –jar GenomeAnalysisTK.jar \

-T RealignerTargetCreator \

-T $ref_fa –o $intervals_file \

-known $known_indels_file(s)

java $jvm_args –jar GenomeAnalysisTK.jar \

-T IndelRealigner \

-R $ref_fa –I $bam_file –o $realigned_bam_file \

-targetIntervals $intervals_file \

-known $known_indels_file(s) \

-LOD 0.4 –model KNOWNS_ONLY –compress 0 –disable_bam_indexing

Base quality scores were then recalibrated using GATK BaseRecalibrator, AnalyzeCovariantes and PrintReads.

java $jvm_args –jar GenomeAnalysisTK.jar \

-T BaseRecalibrator –I $realign_bam –R $ref_fa –o $recalibration_report.grp \

-nct 8 –downsampling_type NONE \

-knownSites $known_sites_file(s)

-cov ReadGroupCovariate –cov QualityScoreCovariate –cov ContextCovariate

java $jvm_args –jar GenomeAnalysisTK.jar \

-T PrintReads –R $ref_fa \

-I $realign_bam –BQSR $recalibration_report.grp \

-o $realign_recal.bam

The realigned and recalibrated BAMs were then passed through samtools to further filter the reads with low quality and reads with template having multiple segments in sequencing, segment unmapped, secondary alignment and not passing filters.

samtools view –f 0xF0C $realign_recal.bam > $bam

2.4 The coverage of whole genome and whole exome sequencing

**3. Variant Calling (SNV)**

We used Atlas5, GATK’s HaplotypeCaller (https://www.broadinstitute.org/gatk/guide/tooldocs/org_broadinstitute_gatk_tools_walkers_haplotypecaller_HaplotypeCaller.php) and GotCloud6 for both whole genome and whole exome sequencing (goSNAP: http://sourceforge.net/p/gosnap/git/ ) in joint calling across the samples. The low quality variants were filtered according to the standard filtering criteria of different callers. We identified 7,214,171 SNPs from 10 whole genome sequenced samples and 81,326 SNPs from 10 whole exome sequenced samples. The average Ti/Tv ratios is 2.08 and 2.26 for whole genome sequencing and whole exome sequencing, respectively. 95.11% whole genome SNPs and 94.02% whole exome SNPs were found in dbSNP141 dataset .

**Table S1. Background information of microarray data obtained from Gene Expression Omnibus.**

| **GEO accession ID** | **Platform** | **Sample type** |
| --- | --- | --- |
| [GSM1955147](https://www.ncbi.nlm.nih.gov/geo/query/acc.cgi?acc=GSM1955147) | GPL570 | intracranial aneurysm1 matched superficial temporal artery |
| [GSM1955148](https://www.ncbi.nlm.nih.gov/geo/query/acc.cgi?acc=GSM1955148) | GPL571 | intracranial aneurysm1 |
| [GSM1955149](https://www.ncbi.nlm.nih.gov/geo/query/acc.cgi?acc=GSM1955149) | GPL572 | intracranial aneurysm2 matched superficial temporal artery |
| [GSM1955150](https://www.ncbi.nlm.nih.gov/geo/query/acc.cgi?acc=GSM1955150) | GPL573 | intracranial aneurysm2 |
| [GSM1955151](https://www.ncbi.nlm.nih.gov/geo/query/acc.cgi?acc=GSM1955151) | GPL574 | intracranial aneurysm3 matched superficial temporal artery |
| [GSM1955152](https://www.ncbi.nlm.nih.gov/geo/query/acc.cgi?acc=GSM1955152) | GPL575 | intracranial aneurysm3 |
| [GSM1955153](https://www.ncbi.nlm.nih.gov/geo/query/acc.cgi?acc=GSM1955153) | GPL576 | intracranial aneurysm4 |
| [GSM1955154](https://www.ncbi.nlm.nih.gov/geo/query/acc.cgi?acc=GSM1955154) | GPL577 | intracranial aneurysm4 matched superficial temporal artery |
| [GSM1955155](https://www.ncbi.nlm.nih.gov/geo/query/acc.cgi?acc=GSM1955155) | GPL578 | intracranial aneurysm5 |
| [GSM1955156](https://www.ncbi.nlm.nih.gov/geo/query/acc.cgi?acc=GSM1955156) | GPL579 | intracranial aneurysm5 matched superficial temporal artery |
| [GSM1955157](https://www.ncbi.nlm.nih.gov/geo/query/acc.cgi?acc=GSM1955157) | GPL580 | intracranial aneurysm6 |
| [GSM1955158](https://www.ncbi.nlm.nih.gov/geo/query/acc.cgi?acc=GSM1955158) | GPL581 | intracranial aneurysm6 matched superficial temporal artery |
| [GSM1955159](https://www.ncbi.nlm.nih.gov/geo/query/acc.cgi?acc=GSM1955159) | GPL582 | intracranial aneurysm7 |
| [GSM1955160](https://www.ncbi.nlm.nih.gov/geo/query/acc.cgi?acc=GSM1955160) | GPL583 | intracranial aneurysm7 matched superficial temporal artery |
| [GSM1955161](https://www.ncbi.nlm.nih.gov/geo/query/acc.cgi?acc=GSM1955161) | GPL584 | intracranial aneurysm8 |
| [GSM1955162](https://www.ncbi.nlm.nih.gov/geo/query/acc.cgi?acc=GSM1955162) | GPL585 | intracranial aneurysm8 matched superficial temporal artery |
| [GSM1955163](https://www.ncbi.nlm.nih.gov/geo/query/acc.cgi?acc=GSM1955163) | GPL586 | intracranial aneurysm9 |
| [GSM1955164](https://www.ncbi.nlm.nih.gov/geo/query/acc.cgi?acc=GSM1955164) | GPL587 | intracranial aneurysm9 matched superficial temporal artery |
| [GSM1955165](https://www.ncbi.nlm.nih.gov/geo/query/acc.cgi?acc=GSM1955165) | GPL588 | intracranial aneurysm10 |
| [GSM1955166](https://www.ncbi.nlm.nih.gov/geo/query/acc.cgi?acc=GSM1955166) | GPL589 | intracranial aneurysm10 matched superficial temporal artery |
| [GSM1955167](https://www.ncbi.nlm.nih.gov/geo/query/acc.cgi?acc=GSM1955167) | GPL590 | intracranial aneurysm11 |
| [GSM1955168](https://www.ncbi.nlm.nih.gov/geo/query/acc.cgi?acc=GSM1955168) | GPL591 | intracranial aneurysm11 matched superficial temporal artery |
| [GSM1955169](https://www.ncbi.nlm.nih.gov/geo/query/acc.cgi?acc=GSM1955169) | GPL592 | intracranial aneurysm12 |
| [GSM1955170](https://www.ncbi.nlm.nih.gov/geo/query/acc.cgi?acc=GSM1955170) | GPL593 | intracranial aneurysm12 matched superficial temporal artery |
| [GSM1955171](https://www.ncbi.nlm.nih.gov/geo/query/acc.cgi?acc=GSM1955171) | GPL594 | intracranial aneurysm13 |
| [GSM1955172](https://www.ncbi.nlm.nih.gov/geo/query/acc.cgi?acc=GSM1955172) | GPL595 | intracranial aneurysm13 matched superficial temporal artery |
| [GSM1955173](https://www.ncbi.nlm.nih.gov/geo/query/acc.cgi?acc=GSM1955173) | GPL596 | intracranial aneurysm14 |
| [GSM1955174](https://www.ncbi.nlm.nih.gov/geo/query/acc.cgi?acc=GSM1955174) | GPL597 | intracranial aneurysm14 matched superficial temporal artery |
| [GSM1955175](https://www.ncbi.nlm.nih.gov/geo/query/acc.cgi?acc=GSM1955175) | GPL598 | intracranial aneurysm15 |
| [GSM1955176](https://www.ncbi.nlm.nih.gov/geo/query/acc.cgi?acc=GSM1955176) | GPL599 | intracranial aneurysm15 matched superficial temporal artery |

**Table S2. Differentially methylated sites in the ADAMTS gene family**

| **ID** | **Gene** | **P** | **t** | **B** | **logFC** | **RANGE_START** | **RANGE_END** | **RANGE_GB** |
| --- | --- | --- | --- | --- | --- | --- | --- | --- |
| cg23737229 | ADAMTS1 | 8.29E-03 | 2.98 | -3.32834 | 1.27E-02 | 28218646 | 28218769 | NC_000021.8 |
| cg23733177 | ADAMTS1 | 3.17E-02 | 2.34 | -4.59586 | 2.86E-02 | 28215972 | 28216095 | NC_000021.8 |
| cg20187011 | ADAMTS1 | 5.97E-03 | 3.13 | -3.01143 | 1.06E-01 | 28214928 | 28215051 | NC_000021.8 |
| cg14124415 | ADAMTS1 | 1.10E-03 | 3.91 | -1.3506 | 8.85E-02 | 28212803 | 28212926 | NC_000021.8 |
| cg01444578 | ADAMTS1 | 1.02E-02 | 2.88 | -3.52624 | 5.46E-02 | 28216559 | 28216682 | NC_000021.8 |
| cg00952789 | ADAMTS1 | 3.60E-03 | 3.36 | -2.52045 | 1.70E-01 | 28215206 | 28215329 | NC_000021.8 |
| cg00472814 | ADAMTS1 | 9.82E-03 | 2.9 | -3.49122 | 5.77E-02 | 28217676 | 28217799 | NC_000021.8 |
| cg27015773 | ADAMTS10 | 2.34E-02 | 2.48 | -4.31499 | 1.64E-01 | 8672609 | 8672732 | NC_000019.9 |
| cg25354248 | ADAMTS10 | 2.04E-03 | 3.62 | -1.96069 | 1.79E-01 | 8676862 | 8676985 | NC_000019.9 |
| cg21661347 | ADAMTS10 | 4.38E-03 | 3.27 | -2.71157 | 5.94E-02 | 8674237 | 8674360 | NC_000019.9 |
| cg19802981 | ADAMTS10 | 1.09E-02 | 2.85 | -3.59064 | 6.44E-02 | 8654808 | 8654931 | NC_000019.9 |
| cg19662846 | ADAMTS10 | 3.89E-04 | 4.38 | -0.31848 | 9.55E-02 | 8650410 | 8650533 | NC_000019.9 |
| cg12845177 | ADAMTS10 | 4.59E-04 | 4.31 | -0.48299 | 4.06E-02 | 8650523 | 8650646 | NC_000019.9 |
| cg12085570 | ADAMTS10 | 2.52E-03 | 3.53 | -2.17137 | 8.86E-02 | 8674501 | 8674624 | NC_000019.9 |
| cg11990296 | ADAMTS10 | 6.30E-03 | 3.11 | -3.06434 | 3.92E-02 | 8651775 | 8651898 | NC_000019.9 |
| cg11590772 | ADAMTS10 | 2.69E-03 | 3.5 | -2.23555 | 1.06E-01 | 8657916 | 8658039 | NC_000019.9 |
| cg09279544 | ADAMTS10 | 3.62E-03 | 3.36 | -2.5243 | 7.37E-02 | 8674388 | 8674511 | NC_000019.9 |
| cg02827007 | ADAMTS10 | 1.66E-02 | -2.67 | -3.94816 | -3.01E-02 | 8661081 | 8661204 | NC_000019.9 |
| cg00420246 | ADAMTS10 | 1.47E-02 | -2.71 | -3.87425 | -2.14E-02 | 8653285 | 8653408 | NC_000019.9 |
| cg26573704 | ADAMTS12 | 1.08E-02 | 2.86 | -3.57996 | 4.67E-02 | 33892223 | 33892346 | NC_000005.9 |
| cg23359363 | ADAMTS12 | 1.98E-04 | -4.69 | 0.35821 | -4.47E-02 | 33764247 | 33764370 | NC_000005.9 |
| cg23236370 | ADAMTS12 | 3.72E-04 | 4.4 | -0.27285 | 2.46E-01 | 33892705 | 33892828 | NC_000005.9 |
| cg21874902 | ADAMTS12 | 2.62E-07 | 8.1 | 7.051471 | 1.29E-01 | 33727007 | 33727130 | NC_000005.9 |
| cg21569398 | ADAMTS12 | 4.73E-04 | 4.29 | -0.5138 | 2.24E-01 | 33855241 | 33855364 | NC_000005.9 |
| cg19641747 | ADAMTS12 | 1.26E-06 | 7.22 | 5.465844 | 2.61E-01 | 33832949 | 33833072 | NC_000005.9 |
| cg18519308 | ADAMTS12 | 9.40E-03 | 2.92 | -3.44952 | 7.61E-02 | 33892621 | 33892744 | NC_000005.9 |
| cg15833353 | ADAMTS12 | 1.62E-02 | -2.66 | -3.96532 | -9.29E-02 | 33807278 | 33807401 | NC_000005.9 |
| cg12917072 | ADAMTS12 | 1.15E-03 | -3.89 | -1.39276 | -3.83E-02 | 33758285 | 33758408 | NC_000005.9 |
| cg10627511 | ADAMTS12 | 9.84E-04 | 3.96 | -1.24213 | 1.74E-01 | 33772916 | 33773039 | NC_000005.9 |
| cg10594543 | ADAMTS12 | 1.67E-02 | -2.65 | -3.99453 | -2.74E-02 | 33649717 | 33649840 | NC_000005.9 |
| cg09747891 | ADAMTS12 | 1.42E-03 | 3.83 | -1.55401 | 2.32E-01 | 33893097 | 33893220 | NC_000005.9 |
| cg08768395 | ADAMTS12 | 1.87E-03 | 3.66 | -1.8746 | 9.44E-02 | 33889461 | 33889584 | NC_000005.9 |
| cg07784793 | ADAMTS12 | 1.83E-05 | 5.83 | 2.756597 | 3.71E-02 | 33794720 | 33794843 | NC_000005.9 |
| cg06893139 | ADAMTS12 | 8.58E-04 | 4.02 | -1.10613 | 2.26E-01 | 33852528 | 33852651 | NC_000005.9 |
| cg06448603 | ADAMTS12 | 1.07E-03 | -3.92 | -1.32805 | -4.41E-02 | 33659084 | 33659207 | NC_000005.9 |
| cg03592903 | ADAMTS12 | 3.14E-03 | -3.43 | -2.38692 | -1.04E-01 | 33737936 | 33738059 | NC_000005.9 |
| cg01990593 | ADAMTS12 | 4.01E-03 | 3.31 | -2.6248 | 1.67E-01 | 33890324 | 33890447 | NC_000005.9 |
| cg14802951 | ADAMTS13 | 1.34E-04 | 4.88 | 0.750835 | 1.15E-01 | 136287409 | 136287532 | NC_000009.11 |
| cg14362312 | ADAMTS13 | 3.16E-02 | 2.34 | -4.59293 | 1.44E-02 | 136294076 | 136294199 | NC_000009.11 |
| cg14206140 | ADAMTS13 | 4.50E-02 | -2.16 | -4.9165 | -4.82E-02 | 136303481 | 136303604 | NC_000009.11 |
| cg14110709 | ADAMTS13 | 1.69E-03 | 3.71 | -1.78038 | 7.36E-02 | 136324375 | 136324498 | NC_000009.11 |
| cg08795752 | ADAMTS13 | 6.37E-05 | 5.23 | 1.497631 | 1.32E-01 | 136293271 | 136293394 | NC_000009.11 |
| cg04674581 | ADAMTS13 | 3.00E-02 | -2.36 | -4.5457 | -2.84E-02 | 136283438 | 136283561 | NC_000009.11 |
| cg00115654 | ADAMTS13 | 6.49E-07 | 7.59 | 6.135166 | 1.45E-01 | 136294920 | 136295043 | NC_000009.11 |
| cg24427089 | ADAMTS14 | 3.43E-02 | 2.31 | -4.62633 | 3.55E-02 | 72432550 | 72432673 | NC_000010.10 |
| cg18886109 | ADAMTS14 | 2.52E-02 | -2.45 | -4.3811 | -9.27E-02 | 72514348 | 72514471 | NC_000010.10 |
| cg16352527 | ADAMTS14 | 3.30E-03 | -3.4 | -2.43477 | -1.74E-01 | 72476178 | 72476301 | NC_000010.10 |
| cg14612544 | ADAMTS14 | 4.81E-02 | 2.14 | -4.9368 | 7.36E-02 | 72432573 | 72432696 | NC_000010.10 |
| cg10975049 | ADAMTS14 | 3.56E-02 | 2.28 | -4.70245 | 5.03E-02 | 72432542 | 72432665 | NC_000010.10 |
| cg10445453 | ADAMTS14 | 4.98E-02 | 2.11 | -5.00848 | 5.07E-02 | 72432540 | 72432663 | NC_000010.10 |
| cg02503850 | ADAMTS14 | 1.83E-02 | 2.6 | -4.08443 | 8.48E-02 | 72432552 | 72432675 | NC_000010.10 |
| cg23214308 | ADAMTS15 | 3.35E-02 | 2.31 | -4.64636 | 1.90E-02 | 130318865 | 130318988 | NC_000011.9 |
| cg19333883 | ADAMTS15 | 1.89E-04 | 4.71 | 0.402612 | 9.94E-02 | 130320219 | 130320342 | NC_000011.9 |
| cg12444117 | ADAMTS15 | 4.13E-03 | 3.3 | -2.65305 | 8.31E-02 | 130319691 | 130319814 | NC_000011.9 |
| cg12007053 | ADAMTS15 | 2.48E-03 | 3.53 | -2.15406 | 9.96E-02 | 130319736 | 130319859 | NC_000011.9 |
| cg10773249 | ADAMTS15 | 6.80E-03 | -3.07 | -3.1375 | -5.02E-02 | 130343309 | 130343432 | NC_000011.9 |
| cg10249997 | ADAMTS15 | 2.57E-02 | 2.44 | -4.40103 | 7.16E-02 | 130318055 | 130318178 | NC_000011.9 |
| cg02554089 | ADAMTS15 | 2.56E-02 | -2.44 | -4.39733 | -1.41E-02 | 130341116 | 130341239 | NC_000011.9 |
| cg26868514 | ADAMTS16 | 5.80E-03 | -3.14 | -2.98414 | -2.90E-02 | 5178224 | 5178347 | NC_000005.9 |
| cg24030232 | ADAMTS16 | 2.04E-03 | 3.62 | -1.96415 | 6.79E-02 | 5252567 | 5252690 | NC_000005.9 |
| cg22983903 | ADAMTS16 | 1.82E-02 | 2.62 | -4.03547 | 4.32E-02 | 5140935 | 5141058 | NC_000005.9 |
| cg22562853 | ADAMTS16 | 1.12E-04 | -4.96 | 0.928268 | -5.39E-02 | 5288047 | 5288170 | NC_000005.9 |
| cg21926451 | ADAMTS16 | 3.23E-02 | -2.33 | -4.61367 | -1.22E-02 | 5288241 | 5288364 | NC_000005.9 |
| cg19813025 | ADAMTS16 | 3.07E-03 | 3.44 | -2.36434 | 9.71E-02 | 5139643 | 5139766 | NC_000005.9 |
| cg17760878 | ADAMTS16 | 4.62E-02 | -2.15 | -4.93963 | -1.84E-02 | 5303460 | 5303583 | NC_000005.9 |
| cg17627328 | ADAMTS16 | 1.14E-05 | -6.07 | 3.23194 | -1.80E-01 | 5146343 | 5146466 | NC_000005.9 |
| cg16670809 | ADAMTS16 | 1.54E-02 | 2.69 | -3.91776 | 8.66E-02 | 5139797 | 5139920 | NC_000005.9 |
| cg16508480 | ADAMTS16 | 2.19E-02 | 2.52 | -4.25258 | 7.84E-02 | 5139874 | 5139997 | NC_000005.9 |
| cg15411554 | ADAMTS16 | 2.07E-04 | -4.67 | 0.310916 | -1.39E-01 | 5199806 | 5199929 | NC_000005.9 |
| cg15048991 | ADAMTS16 | 3.54E-02 | 2.28 | -4.69738 | 4.74E-02 | 5139866 | 5139989 | NC_000005.9 |
| cg14521614 | ADAMTS16 | 2.50E-05 | -5.68 | 2.441978 | -6.03E-02 | 5287980 | 5288103 | NC_000005.9 |
| cg10561103 | ADAMTS16 | 2.04E-02 | -2.55 | -4.18314 | -1.84E-02 | 5239933 | 5240056 | NC_000005.9 |
| cg09456340 | ADAMTS16 | 1.34E-02 | -2.75 | -3.79028 | -2.08E-02 | 5288296 | 5288419 | NC_000005.9 |
| cg06623930 | ADAMTS16 | 3.44E-07 | -7.95 | 6.778721 | -1.36E-01 | 5306038 | 5306161 | NC_000005.9 |
| cg06434454 | ADAMTS16 | 2.90E-04 | -4.52 | -0.02583 | -1.34E-01 | 5146320 | 5146443 | NC_000005.9 |
| cg04136610 | ADAMTS16 | 3.38E-02 | 2.3 | -4.6556 | 5.70E-02 | 5139878 | 5140001 | NC_000005.9 |
| cg03209854 | ADAMTS16 | 5.82E-03 | 3.14 | -2.98763 | 8.55E-02 | 5140003 | 5140126 | NC_000005.9 |
| cg02109699 | ADAMTS16 | 1.80E-07 | -8.33 | 7.431493 | -1.46E-01 | 5234710 | 5234833 | NC_000005.9 |
| cg01881590 | ADAMTS16 | 1.32E-04 | -4.88 | 0.764081 | -3.55E-02 | 5302219 | 5302342 | NC_000005.9 |
| cg00127167 | ADAMTS16 | 1.10E-02 | 2.85 | -3.59648 | 1.04E-01 | 5140029 | 5140152 | NC_000005.9 |
| cg00087067 | ADAMTS16 | 1.60E-06 | -7.09 | 5.22443 | -1.32E-01 | 5265509 | 5265632 | NC_000005.9 |
| cg27533244 | ADAMTS17 | 6.65E-04 | 4.14 | -0.85278 | 7.90E-02 | 100537304 | 100537427 | NC_000015.9 |
| cg27511525 | ADAMTS17 | 5.19E-04 | 4.25 | -0.60635 | 1.76E-01 | 100642482 | 100642605 | NC_000015.9 |
| cg26768318 | ADAMTS17 | 1.16E-02 | -2.82 | -3.64907 | -2.29E-02 | 100874800 | 100874923 | NC_000015.9 |
| cg26589251 | ADAMTS17 | 3.26E-02 | -2.32 | -4.62262 | -1.97E-02 | 100786492 | 100786615 | NC_000015.9 |
| cg24368226 | ADAMTS17 | 4.48E-03 | -3.26 | -2.73267 | -9.19E-02 | 100731456 | 100731579 | NC_000015.9 |
| cg23991388 | ADAMTS17 | 4.31E-05 | -5.41 | 1.890333 | -1.32E-01 | 100654760 | 100654883 | NC_000015.9 |
| cg21838773 | ADAMTS17 | 3.57E-03 | -3.37 | -2.51223 | -9.53E-02 | 100538145 | 100538268 | NC_000015.9 |
| cg21314318 | ADAMTS17 | 4.59E-02 | 2.15 | -4.93544 | 7.46E-02 | 100651510 | 100651633 | NC_000015.9 |
| cg19971025 | ADAMTS17 | 1.36E-02 | -2.74 | -3.80465 | -2.61E-02 | 100739896 | 100740019 | NC_000015.9 |
| cg18747283 | ADAMTS17 | 1.11E-02 | -2.84 | -3.60956 | -1.74E-02 | 100664651 | 100664774 | NC_000015.9 |
| cg18586470 | ADAMTS17 | 3.92E-02 | 2.23 | -4.79182 | 8.85E-03 | 100882540 | 100882663 | NC_000015.9 |
| cg18471471 | ADAMTS17 | 3.17E-04 | 4.48 | -0.11461 | 7.47E-02 | 100537686 | 100537809 | NC_000015.9 |
| cg13144432 | ADAMTS17 | 4.72E-02 | -2.14 | -4.95989 | -1.96E-02 | 100513968 | 100514091 | NC_000015.9 |
| cg11107116 | ADAMTS17 | 2.16E-02 | 2.53 | -4.23661 | 3.99E-02 | 100804081 | 100804204 | NC_000015.9 |
| cg09918751 | ADAMTS17 | 2.78E-02 | -2.4 | -4.47457 | -3.03E-02 | 100517450 | 100517573 | NC_000015.9 |
| cg07969419 | ADAMTS17 | 3.18E-03 | 3.45 | -2.34761 | 4.18E-02 | 100880411 | 100880534 | NC_000015.9 |
| cg07015368 | ADAMTS17 | 6.03E-05 | 5.25 | 1.55204 | 1.21E-01 | 100537761 | 100537884 | NC_000015.9 |
| cg05989248 | ADAMTS17 | 2.36E-02 | -2.48 | -4.32273 | -2.27E-02 | 100530651 | 100530774 | NC_000015.9 |
| cg05404715 | ADAMTS17 | 6.22E-03 | -3.11 | -3.05163 | -2.81E-02 | 100516580 | 100516703 | NC_000015.9 |
| cg05129489 | ADAMTS17 | 2.72E-02 | -2.41 | -4.45266 | -3.12E-02 | 100664728 | 100664851 | NC_000015.9 |
| cg05027458 | ADAMTS17 | 2.78E-02 | 2.4 | -4.47526 | 1.51E-02 | 100882165 | 100882288 | NC_000015.9 |
| cg04622200 | ADAMTS17 | 4.34E-05 | -5.41 | 1.885018 | -1.41E-01 | 100654734 | 100654857 | NC_000015.9 |
| cg03300787 | ADAMTS17 | 1.79E-03 | -3.68 | -1.83351 | -6.63E-02 | 100533202 | 100533325 | NC_000015.9 |
| cg02489449 | ADAMTS17 | 1.57E-02 | -2.69 | -3.89467 | -1.25E-01 | 100524428 | 100524551 | NC_000015.9 |
| cg00149679 | ADAMTS17 | 1.20E-02 | -2.81 | -3.67882 | -6.82E-02 | 100733818 | 100733941 | NC_000015.9 |
| cg27608031 | ADAMTS18 | 7.10E-03 | 3.05 | -3.17902 | 8.58E-02 | 77353824 | 77353947 | NC_000016.9 |
| cg05496543 | ADAMTS18 | 2.99E-02 | -2.37 | -4.54012 | -6.78E-02 | 77467409 | 77467532 | NC_000016.9 |
| cg05485250 | ADAMTS18 | 5.32E-04 | 4.24 | -0.63108 | 7.96E-02 | 77465215 | 77465338 | NC_000016.9 |
| cg05178576 | ADAMTS18 | 4.83E-02 | 2.12 | -4.97998 | 2.46E-02 | 77468486 | 77468609 | NC_000016.9 |
| cg05007098 | ADAMTS18 | 4.21E-03 | -3.29 | -2.67126 | -6.47E-02 | 77322779 | 77322902 | NC_000016.9 |
| cg03983058 | ADAMTS18 | 1.10E-02 | 2.84 | -3.60351 | 3.64E-02 | 77369724 | 77369847 | NC_000016.9 |
| cg03238797 | ADAMTS18 | 5.29E-03 | 3.19 | -2.89372 | 3.26E-02 | 77468893 | 77469016 | NC_000016.9 |
| cg02240171 | ADAMTS18 | 3.27E-02 | -2.32 | -4.6244 | -4.88E-02 | 77437748 | 77437871 | NC_000016.9 |
| cg02186542 | ADAMTS18 | 7.06E-03 | 3.08 | -3.12729 | 6.11E-02 | 77469167 | 77469290 | NC_000016.9 |
| cg02084561 | ADAMTS18 | 1.87E-04 | 4.72 | 0.415963 | 6.95E-02 | 77317555 | 77317678 | NC_000016.9 |
| cg01737667 | ADAMTS18 | 1.28E-03 | -3.83 | -1.50671 | -6.28E-02 | 77397865 | 77397988 | NC_000016.9 |
| cg01407406 | ADAMTS18 | 1.38E-02 | 2.74 | -3.8148 | 6.37E-02 | 77468277 | 77468400 | NC_000016.9 |
| cg25391681 | ADAMTS19 | 2.71E-02 | -2.41 | -4.44876 | -2.98E-02 | 128930873 | 128930996 | NC_000005.9 |
| cg25309017 | ADAMTS19 | 2.48E-02 | 2.46 | -4.36908 | 2.35E-02 | 128796266 | 128796389 | NC_000005.9 |
| cg22813290 | ADAMTS19 | 6.33E-03 | -3.1 | -3.06891 | -2.82E-02 | 129074344 | 129074467 | NC_000005.9 |
| cg20776240 | ADAMTS19 | 1.32E-02 | 2.76 | -3.77431 | 1.62E-02 | 128797307 | 128797430 | NC_000005.9 |
| cg15188268 | ADAMTS19 | 1.03E-02 | 2.88 | -3.53371 | 5.98E-02 | 128795183 | 128795306 | NC_000005.9 |
| cg13701109 | ADAMTS19 | 1.14E-02 | 2.85 | -3.58477 | 5.31E-02 | 128795472 | 128795595 | NC_000005.9 |
| cg07781332 | ADAMTS19 | 3.66E-03 | 3.36 | -2.53519 | 2.66E-02 | 128796696 | 128796819 | NC_000005.9 |
| cg06484432 | ADAMTS19 | 6.31E-08 | -8.96 | 8.489086 | -1.42E-01 | 128801407 | 128801530 | NC_000005.9 |
| cg05211662 | ADAMTS19 | 1.64E-03 | 3.72 | -1.74896 | 6.59E-02 | 128795605 | 128795728 | NC_000005.9 |
| cg00967489 | ADAMTS19 | 3.78E-02 | 2.25 | -4.75632 | 7.49E-02 | 128992882 | 128993005 | NC_000005.9 |
| cg27054655 | ADAMTS2 | 1.91E-02 | -2.58 | -4.12495 | -6.50E-02 | 178772969 | 178773092 | NC_000005.9 |
| cg26650846 | ADAMTS2 | 5.36E-03 | -3.18 | -2.9074 | -1.72E-02 | 178772782 | 178772905 | NC_000005.9 |
| cg25930161 | ADAMTS2 | 3.90E-03 | -3.33 | -2.59797 | -3.09E-02 | 178551993 | 178552116 | NC_000005.9 |
| cg25131079 | ADAMTS2 | 6.02E-06 | 6.39 | 3.881198 | 1.50E-01 | 178712051 | 178712174 | NC_000005.9 |
| cg25010832 | ADAMTS2 | 2.97E-02 | -2.37 | -4.53542 | -1.88E-02 | 178634615 | 178634738 | NC_000005.9 |
| cg21901223 | ADAMTS2 | 2.15E-03 | 3.6 | -2.01568 | 8.04E-02 | 178647332 | 178647455 | NC_000005.9 |
| cg21747876 | ADAMTS2 | 1.54E-02 | -2.69 | -3.91729 | -1.47E-02 | 178687984 | 178688107 | NC_000005.9 |
| cg21183664 | ADAMTS2 | 3.60E-04 | 4.42 | -0.24119 | 1.51E-01 | 178594526 | 178594649 | NC_000005.9 |
| cg21052104 | ADAMTS2 | 3.83E-05 | 5.47 | 2.009374 | 1.82E-01 | 178712636 | 178712759 | NC_000005.9 |
| cg20422099 | ADAMTS2 | 3.69E-02 | 2.27 | -4.69373 | 5.20E-02 | 178771393 | 178771516 | NC_000005.9 |
| cg19935909 | ADAMTS2 | 2.25E-02 | -2.51 | -4.27516 | -2.52E-02 | 178759017 | 178759140 | NC_000005.9 |
| cg19489885 | ADAMTS2 | 2.89E-02 | -2.38 | -4.50956 | -8.70E-02 | 178684416 | 178684539 | NC_000005.9 |
| cg18720326 | ADAMTS2 | 5.25E-06 | 6.46 | 4.020481 | 2.34E-01 | 178712084 | 178712207 | NC_000005.9 |
| cg18299793 | ADAMTS2 | 3.29E-03 | -3.4 | -2.43172 | -2.20E-02 | 178651519 | 178651642 | NC_000005.9 |
| cg18275958 | ADAMTS2 | 1.42E-02 | -2.74 | -3.79717 | -4.61E-02 | 178736443 | 178736566 | NC_000005.9 |
| cg18045155 | ADAMTS2 | 1.87E-06 | -7.01 | 5.066049 | -1.22E-01 | 178767472 | 178767595 | NC_000005.9 |
| cg17925174 | ADAMTS2 | 1.51E-03 | -3.76 | -1.66403 | -5.65E-02 | 178614909 | 178615032 | NC_000005.9 |
| cg17359265 | ADAMTS2 | 1.87E-04 | 4.72 | 0.41664 | 9.28E-02 | 178567126 | 178567249 | NC_000005.9 |
| cg17332422 | ADAMTS2 | 6.02E-03 | -3.13 | -3.01984 | -2.34E-02 | 178658477 | 178658600 | NC_000005.9 |
| cg17212277 | ADAMTS2 | 6.00E-05 | -5.26 | 1.558126 | -6.03E-02 | 178660962 | 178661085 | NC_000005.9 |
| cg16604066 | ADAMTS2 | 2.87E-02 | -2.39 | -4.50326 | -1.27E-02 | 178680976 | 178681099 | NC_000005.9 |
| cg15906761 | ADAMTS2 | 2.59E-02 | 2.44 | -4.40919 | 7.18E-02 | 178623035 | 178623158 | NC_000005.9 |
| cg15726814 | ADAMTS2 | 8.51E-03 | -2.97 | -3.35415 | -3.45E-02 | 178594990 | 178595113 | NC_000005.9 |
| cg14830846 | ADAMTS2 | 1.95E-02 | -2.57 | -4.14085 | -2.37E-02 | 178763293 | 178763416 | NC_000005.9 |
| cg13909534 | ADAMTS2 | 4.42E-02 | 2.17 | -4.90092 | 1.48E-02 | 178772372 | 178772495 | NC_000005.9 |
| cg13380103 | ADAMTS2 | 4.88E-07 | -7.75 | 6.424432 | -1.02E-01 | 178770629 | 178770752 | NC_000005.9 |
| cg13041355 | ADAMTS2 | 1.15E-02 | -2.82 | -3.64612 | -2.66E-02 | 178622080 | 178622203 | NC_000005.9 |
| cg11791960 | ADAMTS2 | 9.81E-06 | 6.15 | 3.386856 | 9.36E-02 | 178667819 | 178667942 | NC_000005.9 |
| cg11200794 | ADAMTS2 | 3.34E-02 | -2.31 | -4.64329 | -1.15E-01 | 178684000 | 178684123 | NC_000005.9 |
| cg10997906 | ADAMTS2 | 1.37E-02 | 2.74 | -3.80863 | 1.23E-01 | 178753803 | 178753926 | NC_000005.9 |
| cg10213542 | ADAMTS2 | 1.16E-03 | 3.88 | -1.40923 | 1.50E-01 | 178692728 | 178692851 | NC_000005.9 |
| cg09540085 | ADAMTS2 | 2.05E-02 | -2.55 | -4.1917 | -2.54E-02 | 178650512 | 178650635 | NC_000005.9 |
| cg08999896 | ADAMTS2 | 2.00E-02 | -2.56 | -4.16721 | -6.87E-02 | 178685787 | 178685910 | NC_000005.9 |
| cg08902683 | ADAMTS2 | 6.39E-04 | -4.15 | -0.81434 | -4.37E-02 | 178748193 | 178748316 | NC_000005.9 |
| cg06495631 | ADAMTS2 | 3.31E-03 | 3.4 | -2.4382 | 1.47E-01 | 178692806 | 178692929 | NC_000005.9 |
| cg05656566 | ADAMTS2 | 2.55E-03 | 3.52 | -2.1828 | 7.60E-02 | 178593924 | 178594047 | NC_000005.9 |
| cg04184432 | ADAMTS2 | 1.59E-03 | -3.74 | -1.71635 | -6.09E-02 | 178622120 | 178622243 | NC_000005.9 |
| cg03259333 | ADAMTS2 | 3.16E-03 | -3.42 | -2.39088 | -1.33E-01 | 178592153 | 178592276 | NC_000005.9 |
| cg02946518 | ADAMTS2 | 1.44E-02 | -2.72 | -3.8586 | -1.24E-01 | 178683834 | 178683957 | NC_000005.9 |
| cg02497046 | ADAMTS2 | 2.24E-04 | -4.64 | 0.235545 | -1.13E-01 | 178636918 | 178637041 | NC_000005.9 |
| cg01791000 | ADAMTS2 | 1.66E-03 | -3.72 | -1.76193 | -3.27E-02 | 178687780 | 178687903 | NC_000005.9 |
| cg01231141 | ADAMTS2 | 7.07E-03 | 3.05 | -3.1753 | 1.50E-01 | 178692691 | 178692814 | NC_000005.9 |
| cg00799121 | ADAMTS2 | 4.04E-02 | 2.22 | -4.81816 | 1.27E-01 | 178741283 | 178741406 | NC_000005.9 |
| cg00562312 | ADAMTS2 | 4.49E-02 | 2.16 | -4.91405 | 3.04E-02 | 178752999 | 178753122 | NC_000005.9 |
| cg00096810 | ADAMTS2 | 4.55E-03 | 3.26 | -2.74795 | 9.91E-02 | 178753432 | 178753555 | NC_000005.9 |
| cg24643381 | ADAMTS20 | 3.10E-06 | -6.74 | 4.553948 | -8.64E-02 | 43941378 | 43941501 | NC_000012.11 |
| cg24606791 | ADAMTS20 | 6.54E-03 | 3.09 | -3.09988 | 1.35E-01 | 43946493 | 43946616 | NC_000012.11 |
| cg24116870 | ADAMTS20 | 5.43E-03 | 3.17 | -2.91956 | 1.06E-01 | 43946284 | 43946407 | NC_000012.11 |
| cg21911301 | ADAMTS20 | 1.96E-02 | 2.57 | -4.14899 | 4.41E-02 | 43946518 | 43946641 | NC_000012.11 |
| cg19619405 | ADAMTS20 | 2.71E-02 | 2.41 | -4.45157 | 6.98E-02 | 43945892 | 43946015 | NC_000012.11 |
| cg17459298 | ADAMTS20 | 2.84E-03 | 3.47 | -2.28949 | 4.78E-02 | 43946341 | 43946464 | NC_000012.11 |
| cg11751806 | ADAMTS20 | 9.88E-03 | 2.9 | -3.4969 | 5.54E-02 | 43945680 | 43945803 | NC_000012.11 |
| cg02925039 | ADAMTS20 | 1.57E-02 | 2.68 | -3.93924 | 6.93E-02 | 43945213 | 43945336 | NC_000012.11 |
| cg01345315 | ADAMTS20 | 3.72E-03 | 3.35 | -2.55286 | 9.78E-02 | 43945998 | 43946121 | NC_000012.11 |
| cg14349862 | ADAMTS3 | 1.40E-04 | 4.86 | 0.708603 | 6.05E-02 | 73178545 | 73178668 | NC_000004.11 |
| cg05797623 | ADAMTS3 | 8.53E-04 | 4.02 | -1.10074 | 2.16E-01 | 73178007 | 73178130 | NC_000004.11 |
| cg04812351 | ADAMTS3 | 5.77E-04 | 4.2 | -0.71264 | 2.13E-01 | 73177995 | 73178118 | NC_000004.11 |
| cg01664241 | ADAMTS3 | 5.38E-03 | 3.18 | -2.91121 | 7.32E-02 | 73435425 | 73435548 | NC_000004.11 |
| cg24329783 | ADAMTS4 | 1.56E-02 | 2.68 | -3.93469 | 4.89E-02 | 161160887 | 161161010 | NC_000001.10 |
| cg07354440 | ADAMTS4 | 1.69E-04 | 4.77 | 0.515912 | 2.47E-01 | 161168889 | 161169012 | NC_000001.10 |
| cg20156545 | ADAMTS5 | 4.25E-06 | 6.57 | 4.233858 | 2.58E-01 | 28335020 | 28335143 | NC_000021.8 |
| cg15237494 | ADAMTS5 | 6.09E-03 | 3.12 | -3.03084 | 1.02E-01 | 28339334 | 28339457 | NC_000021.8 |
| cg14740657 | ADAMTS5 | 4.22E-02 | 2.19 | -4.85925 | 6.57E-02 | 28337594 | 28337717 | NC_000021.8 |
| cg13601496 | ADAMTS5 | 4.41E-02 | 2.17 | -4.8973 | 3.21E-02 | 28339487 | 28339610 | NC_000021.8 |
| cg07771160 | ADAMTS5 | 3.76E-06 | 6.64 | 4.356961 | 1.79E-01 | 28335945 | 28336068 | NC_000021.8 |
| cg03294207 | ADAMTS5 | 1.12E-03 | 3.9 | -1.36917 | 1.23E-01 | 28336897 | 28337020 | NC_000021.8 |
| cg03202077 | ADAMTS5 | 8.46E-04 | 4.02 | -1.09316 | 7.66E-02 | 28338836 | 28338959 | NC_000021.8 |
| cg02800810 | ADAMTS5 | 1.88E-02 | 2.59 | -4.10788 | 7.27E-02 | 28340139 | 28340262 | NC_000021.8 |
| cg00646084 | ADAMTS5 | 8.41E-03 | 2.97 | -3.34255 | 9.57E-02 | 28337168 | 28337291 | NC_000021.8 |
| cg25229198 | ADAMTS6 | 1.15E-03 | 3.89 | -1.39498 | 1.21E-01 | 64660684 | 64660807 | NC_000005.9 |
| cg21033632 | ADAMTS6 | 2.39E-04 | -4.61 | 0.170024 | -1.41E-01 | 64486421 | 64486544 | NC_000005.9 |
| cg16580073 | ADAMTS6 | 1.16E-04 | 4.94 | 0.890789 | 1.10E-01 | 64446603 | 64446726 | NC_000005.9 |
| cg00299603 | ADAMTS6 | 1.22E-03 | -3.86 | -1.45411 | -3.15E-02 | 64494334 | 64494457 | NC_000005.9 |
| cg25490185 | ADAMTS7 | 9.87E-03 | -2.9 | -3.49563 | -2.89E-02 | 79094603 | 79094726 | NC_000015.9 |
| cg23506979 | ADAMTS7 | 4.09E-03 | 3.31 | -2.64339 | 1.96E-01 | 79090958 | 79091081 | NC_000015.9 |
| cg22183295 | ADAMTS7 | 4.34E-02 | -2.18 | -4.88308 | -9.65E-03 | 79099845 | 79099968 | NC_000015.9 |
| cg06752398 | ADAMTS7 | 2.06E-02 | -2.55 | -4.19227 | -9.62E-02 | 79053858 | 79053981 | NC_000015.9 |
| cg03762349 | ADAMTS7 | 1.17E-05 | 6.06 | 3.209496 | 9.44E-02 | 79060523 | 79060646 | NC_000015.9 |
| cg23886747 | ADAMTS8 | 1.85E-04 | 4.72 | 0.424834 | 1.64E-01 | 130297197 | 130297320 | NC_000011.9 |
| cg16453560 | ADAMTS8 | 8.65E-03 | 2.96 | -3.36968 | 2.57E-02 | 130298756 | 130298879 | NC_000011.9 |
| cg13464448 | ADAMTS8 | 5.81E-03 | 3.14 | -2.98603 | 3.30E-02 | 130297513 | 130297636 | NC_000011.9 |
| cg12270485 | ADAMTS8 | 1.74E-02 | -2.63 | -4.03695 | -8.90E-02 | 130299947 | 130300070 | NC_000011.9 |
| cg10521851 | ADAMTS8 | 6.42E-03 | 3.1 | -3.08227 | 6.25E-02 | 130297771 | 130297894 | NC_000011.9 |
| cg09134593 | ADAMTS8 | 7.54E-04 | 4.08 | -0.97782 | 6.98E-02 | 130297528 | 130297651 | NC_000011.9 |
| cg08182171 | ADAMTS8 | 6.86E-04 | -4.12 | -0.88401 | -9.34E-02 | 130291156 | 130291279 | NC_000011.9 |
| cg06944693 | ADAMTS8 | 4.20E-02 | -2.19 | -4.85468 | -2.81E-02 | 130299298 | 130299421 | NC_000011.9 |
| cg04268950 | ADAMTS8 | 7.96E-05 | 5.12 | 1.273129 | 1.27E-01 | 130298854 | 130298977 | NC_000011.9 |
| cg02518691 | ADAMTS8 | 1.18E-04 | 4.93 | 0.873593 | 1.67E-01 | 130297149 | 130297272 | NC_000011.9 |
| cg01033938 | ADAMTS8 | 6.44E-03 | 3.1 | -3.0844 | 7.69E-02 | 130298880 | 130299003 | NC_000011.9 |
| cg27203924 | ADAMTS9 | 3.65E-02 | -2.27 | -4.72496 | -3.02E-02 | 64627542 | 64627665 | NC_000003.11 |
| cg26061177 | ADAMTS9 | 1.73E-03 | 3.7 | -1.80224 | 6.24E-02 | 64670562 | 64670685 | NC_000003.11 |
| cg25859972 | ADAMTS9 | 3.05E-04 | 4.49 | -0.07502 | 1.29E-01 | 64670515 | 64670638 | NC_000003.11 |
| cg25581822 | ADAMTS9 | 3.61E-06 | 6.66 | 4.400094 | 1.47E-01 | 64534657 | 64534780 | NC_000003.11 |
| cg25157607 | ADAMTS9 | 2.79E-02 | 2.4 | -4.47671 | 1.31E-02 | 64672708 | 64672831 | NC_000003.11 |
| cg23070026 | ADAMTS9 | 4.81E-04 | 4.28 | -0.5305 | 2.47E-02 | 64672542 | 64672665 | NC_000003.11 |
| cg22177868 | ADAMTS9 | 7.23E-07 | 7.53 | 6.025938 | 1.39E-01 | 64667730 | 64667853 | NC_000003.11 |
| cg22118297 | ADAMTS9 | 9.75E-09 | 1.02E+01 | 10.36397 | 2.78E-01 | 64547310 | 64547433 | NC_000003.11 |
| cg22081832 | ADAMTS9 | 5.88E-04 | 4.19 | -0.73075 | 1.48E-01 | 64670459 | 64670582 | NC_000003.11 |
| cg21938436 | ADAMTS9 | 5.34E-03 | 3.18 | -2.90337 | 1.07E-01 | 64670985 | 64671108 | NC_000003.11 |
| cg21878275 | ADAMTS9 | 2.59E-02 | 2.44 | -4.40927 | 3.99E-02 | 64673501 | 64673624 | NC_000003.11 |
| cg21527616 | ADAMTS9 | 4.41E-10 | 1.24E+01 | 13.43783 | 2.91E-01 | 64547373 | 64547496 | NC_000003.11 |
| cg20180538 | ADAMTS9 | 1.63E-02 | -2.66 | -3.97162 | -3.97E-02 | 64559102 | 64559225 | NC_000003.11 |
| cg14187266 | ADAMTS9 | 7.31E-10 | 1.20E+01 | 12.93932 | 3.31E-01 | 64547346 | 64547469 | NC_000003.11 |
| cg12478384 | ADAMTS9 | 5.12E-03 | 3.26 | -2.7651 | 1.13E-01 | 64671764 | 64671887 | NC_000003.11 |
| cg12187213 | ADAMTS9 | 3.60E-02 | -2.27 | -4.71271 | -2.10E-02 | 64673741 | 64673864 | NC_000003.11 |
| cg11427510 | ADAMTS9 | 3.56E-02 | 2.28 | -4.7032 | 3.59E-02 | 64673495 | 64673618 | NC_000003.11 |
| cg10447977 | ADAMTS9 | 6.25E-06 | 6.37 | 3.842955 | 2.42E-01 | 64598405 | 64598528 | NC_000003.11 |
| cg07891473 | ADAMTS9 | 5.28E-03 | 3.19 | -2.89182 | 6.86E-02 | 64670640 | 64670763 | NC_000003.11 |
| cg07777540 | ADAMTS9 | 3.70E-03 | 3.35 | -2.54728 | 2.16E-02 | 64524911 | 64525034 | NC_000003.11 |
| cg05501868 | ADAMTS9 | 1.01E-02 | 2.88 | -3.52036 | 1.02E-01 | 64516087 | 64516210 | NC_000003.11 |
| cg04421973 | ADAMTS9 | 2.77E-02 | 2.4 | -4.47216 | 4.20E-02 | 64670907 | 64671030 | NC_000003.11 |
| cg04366011 | ADAMTS9 | 9.40E-03 | 2.97 | -3.35728 | 4.95E-02 | 64671477 | 64671600 | NC_000003.11 |
| cg03427905 | ADAMTS9 | 4.25E-03 | 3.29 | -2.68162 | 7.47E-02 | 64671378 | 64671501 | NC_000003.11 |
| cg02810826 | ADAMTS9 | 5.75E-03 | -3.15 | -2.97518 | -6.20E-02 | 64504415 | 64504538 | NC_000003.11 |
| cg02246130 | ADAMTS9 | 1.44E-09 | 1.15E+01 | 12.26737 | 3.15E-01 | 64547152 | 64547275 | NC_000003.11 |
| cg01635742 | ADAMTS9 | 1.40E-05 | -5.97 | 3.030119 | -7.95E-02 | 64561075 | 64561198 | NC_000003.11 |
| cg01424460 | ADAMTS9 | 2.93E-02 | 2.38 | -4.52139 | 2.46E-02 | 64673301 | 64673424 | NC_000003.11 |
| cg00344411 | ADAMTS9 | 8.61E-11 | 1.38E+01 | 15.0353 | 3.98E-01 | 64547108 | 64547231 | NC_000003.11 |
| ch.9.357218F | ADAMTSL1 | 8.55E-04 | 4.07 | -1.04933 | 4.72E-02 | 18618131 | 18618254 | NC_000009.11 |
| cg14502847 | ADAMTSL1 | 2.85E-04 | -4.53 | -0.00656 | -1.41E-01 | 18490009 | 18490132 | NC_000009.11 |
| cg14174232 | ADAMTSL1 | 9.22E-03 | 2.93 | -3.43016 | 2.61E-02 | 18909891 | 18910014 | NC_000009.11 |
| cg14003978 | ADAMTSL1 | 3.16E-04 | 4.48 | -0.11223 | 2.06E-01 | 18862115 | 18862238 | NC_000009.11 |
| cg13468759 | ADAMTSL1 | 1.28E-07 | 8.53 | 7.7743 | 5.61E-02 | 18605667 | 18605790 | NC_000009.11 |
| cg00116234 | ADAMTSL1 | 4.85E-03 | 3.23 | -2.81038 | 3.65E-02 | 18474243 | 18474366 | NC_000009.11 |
| cg21202204 | ADAMTSL2 | 3.53E-06 | -6.67 | 4.421045 | -1.98E-01 | 136398165 | 136398288 | NC_000009.11 |
| cg18783801 | ADAMTSL2 | 9.14E-03 | 2.93 | -3.42237 | 3.39E-02 | 136419751 | 136419874 | NC_000009.11 |
| cg14389669 | ADAMTSL2 | 2.32E-04 | 4.62 | 0.200144 | 9.75E-02 | 136399386 | 136399509 | NC_000009.11 |
| cg14201417 | ADAMTSL2 | 1.16E-02 | 2.82 | -3.65046 | 4.01E-02 | 136400057 | 136400180 | NC_000009.11 |
| cg13872238 | ADAMTSL2 | 1.27E-06 | 7.22 | 5.453845 | 1.78E-01 | 136430793 | 136430916 | NC_000009.11 |
| cg13748845 | ADAMTSL2 | 7.83E-03 | -3 | -3.27359 | -4.04E-02 | 136434290 | 136434413 | NC_000009.11 |
| cg13464321 | ADAMTSL2 | 7.42E-03 | -3.03 | -3.22101 | -9.92E-02 | 136422991 | 136423114 | NC_000009.11 |
| cg13459291 | ADAMTSL2 | 1.14E-02 | -2.83 | -3.63524 | -4.31E-02 | 136433277 | 136433400 | NC_000009.11 |
| cg11896113 | ADAMTSL2 | 3.05E-02 | -2.36 | -4.55944 | -1.67E-02 | 136397740 | 136397863 | NC_000009.11 |
| cg00742898 | ADAMTSL2 | 2.80E-02 | -2.4 | -4.48117 | -2.32E-02 | 136431882 | 136432005 | NC_000009.11 |
| cg18734428 | ADAMTSL3 | 9.23E-03 | 2.93 | -3.43198 | 5.92E-02 | 84322277 | 84322400 | NC_000015.9 |
| cg15324185 | ADAMTSL3 | 1.08E-02 | -2.85 | -3.5815 | -2.49E-02 | 84612808 | 84612931 | NC_000015.9 |
| cg14598478 | ADAMTSL3 | 1.24E-05 | -6.03 | 3.148488 | -1.95E-01 | 84363061 | 84363184 | NC_000015.9 |
| cg14230666 | ADAMTSL3 | 1.88E-02 | 2.59 | -4.10758 | 8.89E-02 | 84322946 | 84323069 | NC_000015.9 |
| cg11611600 | ADAMTSL3 | 1.56E-02 | 2.68 | -3.93073 | 6.44E-02 | 84323154 | 84323277 | NC_000015.9 |
| cg08115297 | ADAMTSL3 | 2.90E-02 | -2.38 | -4.51256 | -2.81E-02 | 84706833 | 84706956 | NC_000015.9 |
| cg05315510 | ADAMTSL3 | 6.07E-04 | -4.18 | -0.76203 | -3.71E-02 | 84491709 | 84491832 | NC_000015.9 |
| cg04138185 | ADAMTSL3 | 1.12E-02 | 2.84 | -3.61308 | 7.37E-02 | 84322584 | 84322707 | NC_000015.9 |
| cg01152302 | ADAMTSL3 | 1.16E-04 | 4.94 | 0.890668 | 1.08E-01 | 84323770 | 84323893 | NC_000015.9 |
| cg27630771 | ADAMTSL4 | 6.62E-04 | 4.14 | -0.8494 | 8.61E-02 | 150533123 | 150533246 | NC_000001.10 |
| cg26875450 | ADAMTSL4 | 2.55E-02 | 2.44 | -4.39265 | 2.95E-02 | 150530531 | 150530654 | NC_000001.10 |
| cg24441324 | ADAMTSL4 | 1.33E-04 | 4.88 | 0.758366 | 2.73E-01 | 150523231 | 150523354 | NC_000001.10 |
| cg21996039 | ADAMTSL4 | 2.08E-03 | -3.61 | -1.98132 | -3.19E-02 | 150525424 | 150525547 | NC_000001.10 |
| cg15107203 | ADAMTSL4 | 1.12E-05 | 6.08 | 3.251178 | 1.74E-01 | 150529170 | 150529293 | NC_000001.10 |
| cg14037218 | ADAMTSL4 | 2.12E-04 | 4.66 | 0.287655 | 1.83E-01 | 150522367 | 150522490 | NC_000001.10 |
| cg12974394 | ADAMTSL4 | 1.54E-04 | 4.81 | 0.607855 | 1.82E-01 | 150522266 | 150522389 | NC_000001.10 |
| cg09622957 | ADAMTSL4 | 1.97E-02 | -2.57 | -4.1511 | -1.54E-02 | 150521190 | 150521313 | NC_000001.10 |
| cg06185850 | ADAMTSL4 | 2.49E-02 | 2.46 | -4.37028 | 2.45E-02 | 150529828 | 150529951 | NC_000001.10 |
| cg01922433 | ADAMTSL4 | 2.21E-04 | 4.64 | 0.24627 | 6.39E-02 | 150529761 | 150529884 | NC_000001.10 |
| cg24892628 | ADAMTSL5 | 1.44E-03 | 3.78 | -1.61653 | 1.52E-01 | 1509841 | 1509964 | NC_000019.9 |
| cg22994198 | ADAMTSL5 | 1.54E-03 | 3.75 | -1.68878 | 7.54E-02 | 1508528 | 1508651 | NC_000019.9 |
| cg17720286 | ADAMTSL5 | 4.71E-05 | -5.37 | 1.801427 | -8.49E-02 | 1513941 | 1514064 | NC_000019.9 |
| cg17147471 | ADAMTSL5 | 3.98E-04 | 4.37 | -0.34257 | 9.43E-02 | 1510584 | 1510707 | NC_000019.9 |
| cg15002904 | ADAMTSL5 | 1.47E-02 | 2.71 | -3.87562 | 8.99E-02 | 1510692 | 1510815 | NC_000019.9 |
| cg13434396 | ADAMTSL5 | 4.44E-05 | 5.4 | 1.862068 | 1.19E-01 | 1508555 | 1508678 | NC_000019.9 |
| cg09791746 | ADAMTSL5 | 3.84E-05 | 5.47 | 2.007186 | 2.31E-01 | 1510494 | 1510617 | NC_000019.9 |
| cg00658405 | ADAMTSL5 | 1.15E-02 | 2.83 | -3.63913 | 9.33E-02 | 1510500 | 1510623 | NC_000019.9 |
| cg17904988 | ADAMTS4 | 5.96E-05 | 5.26 | 1.563915 | 1.79E-01 | 161168451 | 161168574 | NC_000001.10 |
| cg17166812 | ADAMTS4 | 1.64E-02 | 2.66 | -3.98213 | 7.49E-02 | 161169574 | 161169697 | NC_000001.10 |
| cg14448116 | ADAMTS4 | 6.01E-04 | 4.18 | -0.75186 | 8.91E-02 | 161170110 | 161170233 | NC_000001.10 |
| cg14382215 | ADAMTS4 | 2.53E-04 | 4.58 | 0.113222 | 2.14E-01 | 161169007 | 161169130 | NC_000001.10 |
| cg11801851 | ADAMTS4 | 2.88E-05 | 5.61 | 2.299423 | 2.29E-01 | 161169164 | 161169287 | NC_000001.10 |
| cg10493436 | ADAMTS4 | 1.04E-04 | 5 | 1.005824 | 2.64E-01 | 161168957 | 161169080 | NC_000001.10 |
| cg08545169 | ADAMTS4 | 2.60E-05 | 5.66 | 2.399776 | 1.65E-01 | 161169143 | 161169266 | NC_000001.10 |
| cg06760507 | ADAMTS4 | 1.09E-03 | 3.91 | -1.34241 | 1.93E-01 | 161168145 | 161168268 | NC_000001.10 |
| cg04436964 | ADAMTS4 | 4.84E-02 | 2.12 | -4.98285 | 5.36E-02 | 161167745 | 161167868 | NC_000001.10 |
| cg04263215 | ADAMTS4 | 1.48E-04 | 4.83 | 0.64617 | 2.53E-01 | 161168856 | 161168979 | NC_000001.10 |
