# Supplementary figures and images for: Intracranial aneurysm’s association with genetic variants, transcription abnormality, and methylation changes in ADAMTS genes
Source: PeerJ. 2020 Feb 14;8:e8596. doi: 10.7717/peerj.8596 (PMC7025701; doi:10.7717/peerj.8596)

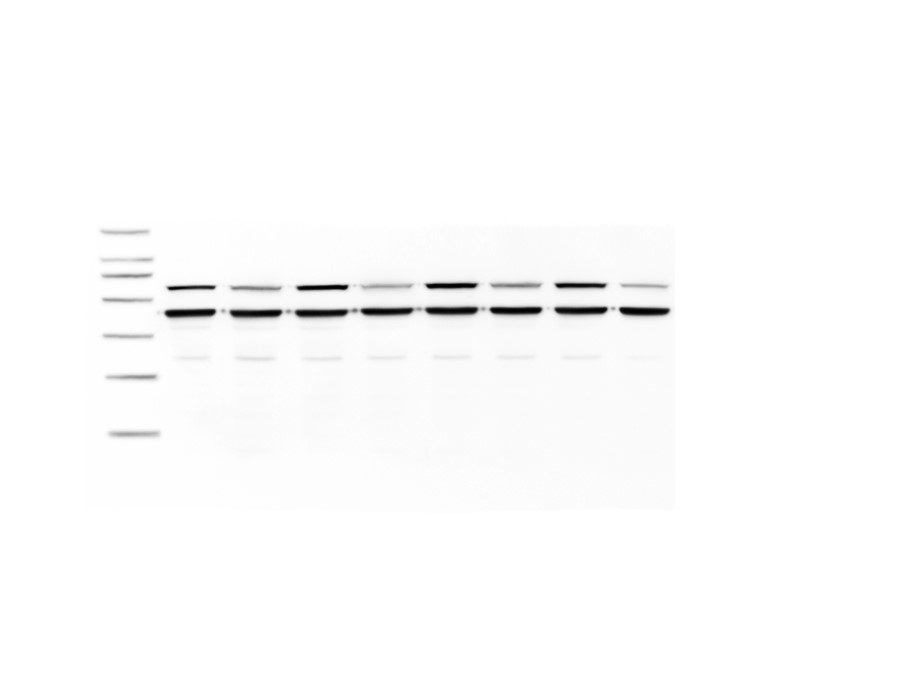

Supplement: Supplemental Information 1 [file peerj-08-8596-s002.zip › original whole image of western-blotting result/Whole image of western-blotting result.jpg]

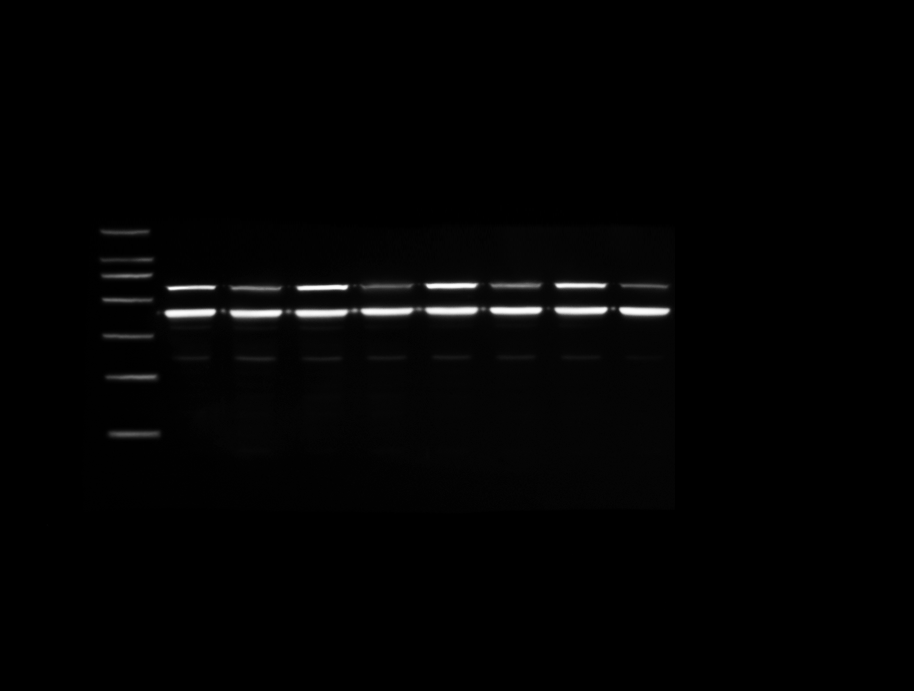

Supplement: Supplemental Information 1 [file peerj-08-8596-s002.zip › original whole image of western-blotting result/original.tif]
