# Supplementary material for: Intracranial aneurysm’s association with genetic variants, transcription abnormality, and methylation changes in ADAMTS genes
Source: PeerJ. 2020 Feb 14;8:e8596. doi: 10.7717/peerj.8596 (PMC7025701; doi:10.7717/peerj.8596)
Supplement: Table S1 [file peerj-08-8596-s003.docx]

**Table S1. Background information of microarray data obtained from Gene Expression Omnibus.**

| **GEO accession ID** | **Platform** | **Sample type** |
| --- | --- | --- |
| [GSM1955147](https://www.ncbi.nlm.nih.gov/geo/query/acc.cgi?acc=GSM1955147) | GPL570 | intracranial aneurysm1 matched superficial temporal artery |
| [GSM1955148](https://www.ncbi.nlm.nih.gov/geo/query/acc.cgi?acc=GSM1955148) | GPL571 | intracranial aneurysm1 |
| [GSM1955149](https://www.ncbi.nlm.nih.gov/geo/query/acc.cgi?acc=GSM1955149) | GPL572 | intracranial aneurysm2 matched superficial temporal artery |
| [GSM1955150](https://www.ncbi.nlm.nih.gov/geo/query/acc.cgi?acc=GSM1955150) | GPL573 | intracranial aneurysm2 |
| [GSM1955151](https://www.ncbi.nlm.nih.gov/geo/query/acc.cgi?acc=GSM1955151) | GPL574 | intracranial aneurysm3 matched superficial temporal artery |
| [GSM1955152](https://www.ncbi.nlm.nih.gov/geo/query/acc.cgi?acc=GSM1955152) | GPL575 | intracranial aneurysm3 |
| [GSM1955153](https://www.ncbi.nlm.nih.gov/geo/query/acc.cgi?acc=GSM1955153) | GPL576 | intracranial aneurysm4 |
| [GSM1955154](https://www.ncbi.nlm.nih.gov/geo/query/acc.cgi?acc=GSM1955154) | GPL577 | intracranial aneurysm4 matched superficial temporal artery |
| [GSM1955155](https://www.ncbi.nlm.nih.gov/geo/query/acc.cgi?acc=GSM1955155) | GPL578 | intracranial aneurysm5 |
| [GSM1955156](https://www.ncbi.nlm.nih.gov/geo/query/acc.cgi?acc=GSM1955156) | GPL579 | intracranial aneurysm5 matched superficial temporal artery |
| [GSM1955157](https://www.ncbi.nlm.nih.gov/geo/query/acc.cgi?acc=GSM1955157) | GPL580 | intracranial aneurysm6 |
| [GSM1955158](https://www.ncbi.nlm.nih.gov/geo/query/acc.cgi?acc=GSM1955158) | GPL581 | intracranial aneurysm6 matched superficial temporal artery |
| [GSM1955159](https://www.ncbi.nlm.nih.gov/geo/query/acc.cgi?acc=GSM1955159) | GPL582 | intracranial aneurysm7 |
| [GSM1955160](https://www.ncbi.nlm.nih.gov/geo/query/acc.cgi?acc=GSM1955160) | GPL583 | intracranial aneurysm7 matched superficial temporal artery |
| [GSM1955161](https://www.ncbi.nlm.nih.gov/geo/query/acc.cgi?acc=GSM1955161) | GPL584 | intracranial aneurysm8 |
| [GSM1955162](https://www.ncbi.nlm.nih.gov/geo/query/acc.cgi?acc=GSM1955162) | GPL585 | intracranial aneurysm8 matched superficial temporal artery |
| [GSM1955163](https://www.ncbi.nlm.nih.gov/geo/query/acc.cgi?acc=GSM1955163) | GPL586 | intracranial aneurysm9 |
| [GSM1955164](https://www.ncbi.nlm.nih.gov/geo/query/acc.cgi?acc=GSM1955164) | GPL587 | intracranial aneurysm9 matched superficial temporal artery |
| [GSM1955165](https://www.ncbi.nlm.nih.gov/geo/query/acc.cgi?acc=GSM1955165) | GPL588 | intracranial aneurysm10 |
| [GSM1955166](https://www.ncbi.nlm.nih.gov/geo/query/acc.cgi?acc=GSM1955166) | GPL589 | intracranial aneurysm10 matched superficial temporal artery |
| [GSM1955167](https://www.ncbi.nlm.nih.gov/geo/query/acc.cgi?acc=GSM1955167) | GPL590 | intracranial aneurysm11 |
| [GSM1955168](https://www.ncbi.nlm.nih.gov/geo/query/acc.cgi?acc=GSM1955168) | GPL591 | intracranial aneurysm11 matched superficial temporal artery |
| [GSM1955169](https://www.ncbi.nlm.nih.gov/geo/query/acc.cgi?acc=GSM1955169) | GPL592 | intracranial aneurysm12 |
| [GSM1955170](https://www.ncbi.nlm.nih.gov/geo/query/acc.cgi?acc=GSM1955170) | GPL593 | intracranial aneurysm12 matched superficial temporal artery |
| [GSM1955171](https://www.ncbi.nlm.nih.gov/geo/query/acc.cgi?acc=GSM1955171) | GPL594 | intracranial aneurysm13 |
| [GSM1955172](https://www.ncbi.nlm.nih.gov/geo/query/acc.cgi?acc=GSM1955172) | GPL595 | intracranial aneurysm13 matched superficial temporal artery |
| [GSM1955173](https://www.ncbi.nlm.nih.gov/geo/query/acc.cgi?acc=GSM1955173) | GPL596 | intracranial aneurysm14 |
| [GSM1955174](https://www.ncbi.nlm.nih.gov/geo/query/acc.cgi?acc=GSM1955174) | GPL597 | intracranial aneurysm14 matched superficial temporal artery |
| [GSM1955175](https://www.ncbi.nlm.nih.gov/geo/query/acc.cgi?acc=GSM1955175) | GPL598 | intracranial aneurysm15 |
| [GSM1955176](https://www.ncbi.nlm.nih.gov/geo/query/acc.cgi?acc=GSM1955176) | GPL599 | intracranial aneurysm15 matched superficial temporal artery |
