# Supplementary material for: Intracranial aneurysm’s association with genetic variants, transcription abnormality, and methylation changes in ADAMTS genes
Source: PeerJ. 2020 Feb 14;8:e8596. doi: 10.7717/peerj.8596 (PMC7025701; doi:10.7717/peerj.8596)
Supplement: Table S2 [file peerj-08-8596-s004.docx]

**Table S2. Differentially methylated sites in the ADAMTS gene family**

| **ID** | **Gene** | **P** | **t** | **B** | **logFC** | **RANGE_START** | **RANGE_END** | **RANGE_GB** |
| --- | --- | --- | --- | --- | --- | --- | --- | --- |
| cg23737229 | ADAMTS1 | 8.29E-03 | 2.98 | -3.32834 | 1.27E-02 | 28218646 | 28218769 | NC_000021.8 |
| cg23733177 | ADAMTS1 | 3.17E-02 | 2.34 | -4.59586 | 2.86E-02 | 28215972 | 28216095 | NC_000021.8 |
| cg20187011 | ADAMTS1 | 5.97E-03 | 3.13 | -3.01143 | 1.06E-01 | 28214928 | 28215051 | NC_000021.8 |
| cg14124415 | ADAMTS1 | 1.10E-03 | 3.91 | -1.3506 | 8.85E-02 | 28212803 | 28212926 | NC_000021.8 |
| cg01444578 | ADAMTS1 | 1.02E-02 | 2.88 | -3.52624 | 5.46E-02 | 28216559 | 28216682 | NC_000021.8 |
| cg00952789 | ADAMTS1 | 3.60E-03 | 3.36 | -2.52045 | 1.70E-01 | 28215206 | 28215329 | NC_000021.8 |
| cg00472814 | ADAMTS1 | 9.82E-03 | 2.9 | -3.49122 | 5.77E-02 | 28217676 | 28217799 | NC_000021.8 |
| cg27015773 | ADAMTS10 | 2.34E-02 | 2.48 | -4.31499 | 1.64E-01 | 8672609 | 8672732 | NC_000019.9 |
| cg25354248 | ADAMTS10 | 2.04E-03 | 3.62 | -1.96069 | 1.79E-01 | 8676862 | 8676985 | NC_000019.9 |
| cg21661347 | ADAMTS10 | 4.38E-03 | 3.27 | -2.71157 | 5.94E-02 | 8674237 | 8674360 | NC_000019.9 |
| cg19802981 | ADAMTS10 | 1.09E-02 | 2.85 | -3.59064 | 6.44E-02 | 8654808 | 8654931 | NC_000019.9 |
| cg19662846 | ADAMTS10 | 3.89E-04 | 4.38 | -0.31848 | 9.55E-02 | 8650410 | 8650533 | NC_000019.9 |
| cg12845177 | ADAMTS10 | 4.59E-04 | 4.31 | -0.48299 | 4.06E-02 | 8650523 | 8650646 | NC_000019.9 |
| cg12085570 | ADAMTS10 | 2.52E-03 | 3.53 | -2.17137 | 8.86E-02 | 8674501 | 8674624 | NC_000019.9 |
| cg11990296 | ADAMTS10 | 6.30E-03 | 3.11 | -3.06434 | 3.92E-02 | 8651775 | 8651898 | NC_000019.9 |
| cg11590772 | ADAMTS10 | 2.69E-03 | 3.5 | -2.23555 | 1.06E-01 | 8657916 | 8658039 | NC_000019.9 |
| cg09279544 | ADAMTS10 | 3.62E-03 | 3.36 | -2.5243 | 7.37E-02 | 8674388 | 8674511 | NC_000019.9 |
| cg02827007 | ADAMTS10 | 1.66E-02 | -2.67 | -3.94816 | -3.01E-02 | 8661081 | 8661204 | NC_000019.9 |
| cg00420246 | ADAMTS10 | 1.47E-02 | -2.71 | -3.87425 | -2.14E-02 | 8653285 | 8653408 | NC_000019.9 |
| cg26573704 | ADAMTS12 | 1.08E-02 | 2.86 | -3.57996 | 4.67E-02 | 33892223 | 33892346 | NC_000005.9 |
| cg23359363 | ADAMTS12 | 1.98E-04 | -4.69 | 0.35821 | -4.47E-02 | 33764247 | 33764370 | NC_000005.9 |
| cg23236370 | ADAMTS12 | 3.72E-04 | 4.4 | -0.27285 | 2.46E-01 | 33892705 | 33892828 | NC_000005.9 |
| cg21874902 | ADAMTS12 | 2.62E-07 | 8.1 | 7.051471 | 1.29E-01 | 33727007 | 33727130 | NC_000005.9 |
| cg21569398 | ADAMTS12 | 4.73E-04 | 4.29 | -0.5138 | 2.24E-01 | 33855241 | 33855364 | NC_000005.9 |
| cg19641747 | ADAMTS12 | 1.26E-06 | 7.22 | 5.465844 | 2.61E-01 | 33832949 | 33833072 | NC_000005.9 |
| cg18519308 | ADAMTS12 | 9.40E-03 | 2.92 | -3.44952 | 7.61E-02 | 33892621 | 33892744 | NC_000005.9 |
| cg15833353 | ADAMTS12 | 1.62E-02 | -2.66 | -3.96532 | -9.29E-02 | 33807278 | 33807401 | NC_000005.9 |
| cg12917072 | ADAMTS12 | 1.15E-03 | -3.89 | -1.39276 | -3.83E-02 | 33758285 | 33758408 | NC_000005.9 |
| cg10627511 | ADAMTS12 | 9.84E-04 | 3.96 | -1.24213 | 1.74E-01 | 33772916 | 33773039 | NC_000005.9 |
| cg10594543 | ADAMTS12 | 1.67E-02 | -2.65 | -3.99453 | -2.74E-02 | 33649717 | 33649840 | NC_000005.9 |
| cg09747891 | ADAMTS12 | 1.42E-03 | 3.83 | -1.55401 | 2.32E-01 | 33893097 | 33893220 | NC_000005.9 |
| cg08768395 | ADAMTS12 | 1.87E-03 | 3.66 | -1.8746 | 9.44E-02 | 33889461 | 33889584 | NC_000005.9 |
| cg07784793 | ADAMTS12 | 1.83E-05 | 5.83 | 2.756597 | 3.71E-02 | 33794720 | 33794843 | NC_000005.9 |
| cg06893139 | ADAMTS12 | 8.58E-04 | 4.02 | -1.10613 | 2.26E-01 | 33852528 | 33852651 | NC_000005.9 |
| cg06448603 | ADAMTS12 | 1.07E-03 | -3.92 | -1.32805 | -4.41E-02 | 33659084 | 33659207 | NC_000005.9 |
| cg03592903 | ADAMTS12 | 3.14E-03 | -3.43 | -2.38692 | -1.04E-01 | 33737936 | 33738059 | NC_000005.9 |
| cg01990593 | ADAMTS12 | 4.01E-03 | 3.31 | -2.6248 | 1.67E-01 | 33890324 | 33890447 | NC_000005.9 |
| cg14802951 | ADAMTS13 | 1.34E-04 | 4.88 | 0.750835 | 1.15E-01 | 136287409 | 136287532 | NC_000009.11 |
| cg14362312 | ADAMTS13 | 3.16E-02 | 2.34 | -4.59293 | 1.44E-02 | 136294076 | 136294199 | NC_000009.11 |
| cg14206140 | ADAMTS13 | 4.50E-02 | -2.16 | -4.9165 | -4.82E-02 | 136303481 | 136303604 | NC_000009.11 |
| cg14110709 | ADAMTS13 | 1.69E-03 | 3.71 | -1.78038 | 7.36E-02 | 136324375 | 136324498 | NC_000009.11 |
| cg08795752 | ADAMTS13 | 6.37E-05 | 5.23 | 1.497631 | 1.32E-01 | 136293271 | 136293394 | NC_000009.11 |
| cg04674581 | ADAMTS13 | 3.00E-02 | -2.36 | -4.5457 | -2.84E-02 | 136283438 | 136283561 | NC_000009.11 |
| cg00115654 | ADAMTS13 | 6.49E-07 | 7.59 | 6.135166 | 1.45E-01 | 136294920 | 136295043 | NC_000009.11 |
| cg24427089 | ADAMTS14 | 3.43E-02 | 2.31 | -4.62633 | 3.55E-02 | 72432550 | 72432673 | NC_000010.10 |
| cg18886109 | ADAMTS14 | 2.52E-02 | -2.45 | -4.3811 | -9.27E-02 | 72514348 | 72514471 | NC_000010.10 |
| cg16352527 | ADAMTS14 | 3.30E-03 | -3.4 | -2.43477 | -1.74E-01 | 72476178 | 72476301 | NC_000010.10 |
| cg14612544 | ADAMTS14 | 4.81E-02 | 2.14 | -4.9368 | 7.36E-02 | 72432573 | 72432696 | NC_000010.10 |
| cg10975049 | ADAMTS14 | 3.56E-02 | 2.28 | -4.70245 | 5.03E-02 | 72432542 | 72432665 | NC_000010.10 |
| cg10445453 | ADAMTS14 | 4.98E-02 | 2.11 | -5.00848 | 5.07E-02 | 72432540 | 72432663 | NC_000010.10 |
| cg02503850 | ADAMTS14 | 1.83E-02 | 2.6 | -4.08443 | 8.48E-02 | 72432552 | 72432675 | NC_000010.10 |
| cg23214308 | ADAMTS15 | 3.35E-02 | 2.31 | -4.64636 | 1.90E-02 | 130318865 | 130318988 | NC_000011.9 |
| cg19333883 | ADAMTS15 | 1.89E-04 | 4.71 | 0.402612 | 9.94E-02 | 130320219 | 130320342 | NC_000011.9 |
| cg12444117 | ADAMTS15 | 4.13E-03 | 3.3 | -2.65305 | 8.31E-02 | 130319691 | 130319814 | NC_000011.9 |
| cg12007053 | ADAMTS15 | 2.48E-03 | 3.53 | -2.15406 | 9.96E-02 | 130319736 | 130319859 | NC_000011.9 |
| cg10773249 | ADAMTS15 | 6.80E-03 | -3.07 | -3.1375 | -5.02E-02 | 130343309 | 130343432 | NC_000011.9 |
| cg10249997 | ADAMTS15 | 2.57E-02 | 2.44 | -4.40103 | 7.16E-02 | 130318055 | 130318178 | NC_000011.9 |
| cg02554089 | ADAMTS15 | 2.56E-02 | -2.44 | -4.39733 | -1.41E-02 | 130341116 | 130341239 | NC_000011.9 |
| cg26868514 | ADAMTS16 | 5.80E-03 | -3.14 | -2.98414 | -2.90E-02 | 5178224 | 5178347 | NC_000005.9 |
| cg24030232 | ADAMTS16 | 2.04E-03 | 3.62 | -1.96415 | 6.79E-02 | 5252567 | 5252690 | NC_000005.9 |
| cg22983903 | ADAMTS16 | 1.82E-02 | 2.62 | -4.03547 | 4.32E-02 | 5140935 | 5141058 | NC_000005.9 |
| cg22562853 | ADAMTS16 | 1.12E-04 | -4.96 | 0.928268 | -5.39E-02 | 5288047 | 5288170 | NC_000005.9 |
| cg21926451 | ADAMTS16 | 3.23E-02 | -2.33 | -4.61367 | -1.22E-02 | 5288241 | 5288364 | NC_000005.9 |
| cg19813025 | ADAMTS16 | 3.07E-03 | 3.44 | -2.36434 | 9.71E-02 | 5139643 | 5139766 | NC_000005.9 |
| cg17760878 | ADAMTS16 | 4.62E-02 | -2.15 | -4.93963 | -1.84E-02 | 5303460 | 5303583 | NC_000005.9 |
| cg17627328 | ADAMTS16 | 1.14E-05 | -6.07 | 3.23194 | -1.80E-01 | 5146343 | 5146466 | NC_000005.9 |
| cg16670809 | ADAMTS16 | 1.54E-02 | 2.69 | -3.91776 | 8.66E-02 | 5139797 | 5139920 | NC_000005.9 |
| cg16508480 | ADAMTS16 | 2.19E-02 | 2.52 | -4.25258 | 7.84E-02 | 5139874 | 5139997 | NC_000005.9 |
| cg15411554 | ADAMTS16 | 2.07E-04 | -4.67 | 0.310916 | -1.39E-01 | 5199806 | 5199929 | NC_000005.9 |
| cg15048991 | ADAMTS16 | 3.54E-02 | 2.28 | -4.69738 | 4.74E-02 | 5139866 | 5139989 | NC_000005.9 |
| cg14521614 | ADAMTS16 | 2.50E-05 | -5.68 | 2.441978 | -6.03E-02 | 5287980 | 5288103 | NC_000005.9 |
| cg10561103 | ADAMTS16 | 2.04E-02 | -2.55 | -4.18314 | -1.84E-02 | 5239933 | 5240056 | NC_000005.9 |
| cg09456340 | ADAMTS16 | 1.34E-02 | -2.75 | -3.79028 | -2.08E-02 | 5288296 | 5288419 | NC_000005.9 |
| cg06623930 | ADAMTS16 | 3.44E-07 | -7.95 | 6.778721 | -1.36E-01 | 5306038 | 5306161 | NC_000005.9 |
| cg06434454 | ADAMTS16 | 2.90E-04 | -4.52 | -0.02583 | -1.34E-01 | 5146320 | 5146443 | NC_000005.9 |
| cg04136610 | ADAMTS16 | 3.38E-02 | 2.3 | -4.6556 | 5.70E-02 | 5139878 | 5140001 | NC_000005.9 |
| cg03209854 | ADAMTS16 | 5.82E-03 | 3.14 | -2.98763 | 8.55E-02 | 5140003 | 5140126 | NC_000005.9 |
| cg02109699 | ADAMTS16 | 1.80E-07 | -8.33 | 7.431493 | -1.46E-01 | 5234710 | 5234833 | NC_000005.9 |
| cg01881590 | ADAMTS16 | 1.32E-04 | -4.88 | 0.764081 | -3.55E-02 | 5302219 | 5302342 | NC_000005.9 |
| cg00127167 | ADAMTS16 | 1.10E-02 | 2.85 | -3.59648 | 1.04E-01 | 5140029 | 5140152 | NC_000005.9 |
| cg00087067 | ADAMTS16 | 1.60E-06 | -7.09 | 5.22443 | -1.32E-01 | 5265509 | 5265632 | NC_000005.9 |
| cg27533244 | ADAMTS17 | 6.65E-04 | 4.14 | -0.85278 | 7.90E-02 | 100537304 | 100537427 | NC_000015.9 |
| cg27511525 | ADAMTS17 | 5.19E-04 | 4.25 | -0.60635 | 1.76E-01 | 100642482 | 100642605 | NC_000015.9 |
| cg26768318 | ADAMTS17 | 1.16E-02 | -2.82 | -3.64907 | -2.29E-02 | 100874800 | 100874923 | NC_000015.9 |
| cg26589251 | ADAMTS17 | 3.26E-02 | -2.32 | -4.62262 | -1.97E-02 | 100786492 | 100786615 | NC_000015.9 |
| cg24368226 | ADAMTS17 | 4.48E-03 | -3.26 | -2.73267 | -9.19E-02 | 100731456 | 100731579 | NC_000015.9 |
| cg23991388 | ADAMTS17 | 4.31E-05 | -5.41 | 1.890333 | -1.32E-01 | 100654760 | 100654883 | NC_000015.9 |
| cg21838773 | ADAMTS17 | 3.57E-03 | -3.37 | -2.51223 | -9.53E-02 | 100538145 | 100538268 | NC_000015.9 |
| cg21314318 | ADAMTS17 | 4.59E-02 | 2.15 | -4.93544 | 7.46E-02 | 100651510 | 100651633 | NC_000015.9 |
| cg19971025 | ADAMTS17 | 1.36E-02 | -2.74 | -3.80465 | -2.61E-02 | 100739896 | 100740019 | NC_000015.9 |
| cg18747283 | ADAMTS17 | 1.11E-02 | -2.84 | -3.60956 | -1.74E-02 | 100664651 | 100664774 | NC_000015.9 |
| cg18586470 | ADAMTS17 | 3.92E-02 | 2.23 | -4.79182 | 8.85E-03 | 100882540 | 100882663 | NC_000015.9 |
| cg18471471 | ADAMTS17 | 3.17E-04 | 4.48 | -0.11461 | 7.47E-02 | 100537686 | 100537809 | NC_000015.9 |
| cg13144432 | ADAMTS17 | 4.72E-02 | -2.14 | -4.95989 | -1.96E-02 | 100513968 | 100514091 | NC_000015.9 |
| cg11107116 | ADAMTS17 | 2.16E-02 | 2.53 | -4.23661 | 3.99E-02 | 100804081 | 100804204 | NC_000015.9 |
| cg09918751 | ADAMTS17 | 2.78E-02 | -2.4 | -4.47457 | -3.03E-02 | 100517450 | 100517573 | NC_000015.9 |
| cg07969419 | ADAMTS17 | 3.18E-03 | 3.45 | -2.34761 | 4.18E-02 | 100880411 | 100880534 | NC_000015.9 |
| cg07015368 | ADAMTS17 | 6.03E-05 | 5.25 | 1.55204 | 1.21E-01 | 100537761 | 100537884 | NC_000015.9 |
| cg05989248 | ADAMTS17 | 2.36E-02 | -2.48 | -4.32273 | -2.27E-02 | 100530651 | 100530774 | NC_000015.9 |
| cg05404715 | ADAMTS17 | 6.22E-03 | -3.11 | -3.05163 | -2.81E-02 | 100516580 | 100516703 | NC_000015.9 |
| cg05129489 | ADAMTS17 | 2.72E-02 | -2.41 | -4.45266 | -3.12E-02 | 100664728 | 100664851 | NC_000015.9 |
| cg05027458 | ADAMTS17 | 2.78E-02 | 2.4 | -4.47526 | 1.51E-02 | 100882165 | 100882288 | NC_000015.9 |
| cg04622200 | ADAMTS17 | 4.34E-05 | -5.41 | 1.885018 | -1.41E-01 | 100654734 | 100654857 | NC_000015.9 |
| cg03300787 | ADAMTS17 | 1.79E-03 | -3.68 | -1.83351 | -6.63E-02 | 100533202 | 100533325 | NC_000015.9 |
| cg02489449 | ADAMTS17 | 1.57E-02 | -2.69 | -3.89467 | -1.25E-01 | 100524428 | 100524551 | NC_000015.9 |
| cg00149679 | ADAMTS17 | 1.20E-02 | -2.81 | -3.67882 | -6.82E-02 | 100733818 | 100733941 | NC_000015.9 |
| cg27608031 | ADAMTS18 | 7.10E-03 | 3.05 | -3.17902 | 8.58E-02 | 77353824 | 77353947 | NC_000016.9 |
| cg05496543 | ADAMTS18 | 2.99E-02 | -2.37 | -4.54012 | -6.78E-02 | 77467409 | 77467532 | NC_000016.9 |
| cg05485250 | ADAMTS18 | 5.32E-04 | 4.24 | -0.63108 | 7.96E-02 | 77465215 | 77465338 | NC_000016.9 |
| cg05178576 | ADAMTS18 | 4.83E-02 | 2.12 | -4.97998 | 2.46E-02 | 77468486 | 77468609 | NC_000016.9 |
| cg05007098 | ADAMTS18 | 4.21E-03 | -3.29 | -2.67126 | -6.47E-02 | 77322779 | 77322902 | NC_000016.9 |
| cg03983058 | ADAMTS18 | 1.10E-02 | 2.84 | -3.60351 | 3.64E-02 | 77369724 | 77369847 | NC_000016.9 |
| cg03238797 | ADAMTS18 | 5.29E-03 | 3.19 | -2.89372 | 3.26E-02 | 77468893 | 77469016 | NC_000016.9 |
| cg02240171 | ADAMTS18 | 3.27E-02 | -2.32 | -4.6244 | -4.88E-02 | 77437748 | 77437871 | NC_000016.9 |
| cg02186542 | ADAMTS18 | 7.06E-03 | 3.08 | -3.12729 | 6.11E-02 | 77469167 | 77469290 | NC_000016.9 |
| cg02084561 | ADAMTS18 | 1.87E-04 | 4.72 | 0.415963 | 6.95E-02 | 77317555 | 77317678 | NC_000016.9 |
| cg01737667 | ADAMTS18 | 1.28E-03 | -3.83 | -1.50671 | -6.28E-02 | 77397865 | 77397988 | NC_000016.9 |
| cg01407406 | ADAMTS18 | 1.38E-02 | 2.74 | -3.8148 | 6.37E-02 | 77468277 | 77468400 | NC_000016.9 |
| cg25391681 | ADAMTS19 | 2.71E-02 | -2.41 | -4.44876 | -2.98E-02 | 128930873 | 128930996 | NC_000005.9 |
| cg25309017 | ADAMTS19 | 2.48E-02 | 2.46 | -4.36908 | 2.35E-02 | 128796266 | 128796389 | NC_000005.9 |
| cg22813290 | ADAMTS19 | 6.33E-03 | -3.1 | -3.06891 | -2.82E-02 | 129074344 | 129074467 | NC_000005.9 |
| cg20776240 | ADAMTS19 | 1.32E-02 | 2.76 | -3.77431 | 1.62E-02 | 128797307 | 128797430 | NC_000005.9 |
| cg15188268 | ADAMTS19 | 1.03E-02 | 2.88 | -3.53371 | 5.98E-02 | 128795183 | 128795306 | NC_000005.9 |
| cg13701109 | ADAMTS19 | 1.14E-02 | 2.85 | -3.58477 | 5.31E-02 | 128795472 | 128795595 | NC_000005.9 |
| cg07781332 | ADAMTS19 | 3.66E-03 | 3.36 | -2.53519 | 2.66E-02 | 128796696 | 128796819 | NC_000005.9 |
| cg06484432 | ADAMTS19 | 6.31E-08 | -8.96 | 8.489086 | -1.42E-01 | 128801407 | 128801530 | NC_000005.9 |
| cg05211662 | ADAMTS19 | 1.64E-03 | 3.72 | -1.74896 | 6.59E-02 | 128795605 | 128795728 | NC_000005.9 |
| cg00967489 | ADAMTS19 | 3.78E-02 | 2.25 | -4.75632 | 7.49E-02 | 128992882 | 128993005 | NC_000005.9 |
| cg27054655 | ADAMTS2 | 1.91E-02 | -2.58 | -4.12495 | -6.50E-02 | 178772969 | 178773092 | NC_000005.9 |
| cg26650846 | ADAMTS2 | 5.36E-03 | -3.18 | -2.9074 | -1.72E-02 | 178772782 | 178772905 | NC_000005.9 |
| cg25930161 | ADAMTS2 | 3.90E-03 | -3.33 | -2.59797 | -3.09E-02 | 178551993 | 178552116 | NC_000005.9 |
| cg25131079 | ADAMTS2 | 6.02E-06 | 6.39 | 3.881198 | 1.50E-01 | 178712051 | 178712174 | NC_000005.9 |
| cg25010832 | ADAMTS2 | 2.97E-02 | -2.37 | -4.53542 | -1.88E-02 | 178634615 | 178634738 | NC_000005.9 |
| cg21901223 | ADAMTS2 | 2.15E-03 | 3.6 | -2.01568 | 8.04E-02 | 178647332 | 178647455 | NC_000005.9 |
| cg21747876 | ADAMTS2 | 1.54E-02 | -2.69 | -3.91729 | -1.47E-02 | 178687984 | 178688107 | NC_000005.9 |
| cg21183664 | ADAMTS2 | 3.60E-04 | 4.42 | -0.24119 | 1.51E-01 | 178594526 | 178594649 | NC_000005.9 |
| cg21052104 | ADAMTS2 | 3.83E-05 | 5.47 | 2.009374 | 1.82E-01 | 178712636 | 178712759 | NC_000005.9 |
| cg20422099 | ADAMTS2 | 3.69E-02 | 2.27 | -4.69373 | 5.20E-02 | 178771393 | 178771516 | NC_000005.9 |
| cg19935909 | ADAMTS2 | 2.25E-02 | -2.51 | -4.27516 | -2.52E-02 | 178759017 | 178759140 | NC_000005.9 |
| cg19489885 | ADAMTS2 | 2.89E-02 | -2.38 | -4.50956 | -8.70E-02 | 178684416 | 178684539 | NC_000005.9 |
| cg18720326 | ADAMTS2 | 5.25E-06 | 6.46 | 4.020481 | 2.34E-01 | 178712084 | 178712207 | NC_000005.9 |
| cg18299793 | ADAMTS2 | 3.29E-03 | -3.4 | -2.43172 | -2.20E-02 | 178651519 | 178651642 | NC_000005.9 |
| cg18275958 | ADAMTS2 | 1.42E-02 | -2.74 | -3.79717 | -4.61E-02 | 178736443 | 178736566 | NC_000005.9 |
| cg18045155 | ADAMTS2 | 1.87E-06 | -7.01 | 5.066049 | -1.22E-01 | 178767472 | 178767595 | NC_000005.9 |
| cg17925174 | ADAMTS2 | 1.51E-03 | -3.76 | -1.66403 | -5.65E-02 | 178614909 | 178615032 | NC_000005.9 |
| cg17359265 | ADAMTS2 | 1.87E-04 | 4.72 | 0.41664 | 9.28E-02 | 178567126 | 178567249 | NC_000005.9 |
| cg17332422 | ADAMTS2 | 6.02E-03 | -3.13 | -3.01984 | -2.34E-02 | 178658477 | 178658600 | NC_000005.9 |
| cg17212277 | ADAMTS2 | 6.00E-05 | -5.26 | 1.558126 | -6.03E-02 | 178660962 | 178661085 | NC_000005.9 |
| cg16604066 | ADAMTS2 | 2.87E-02 | -2.39 | -4.50326 | -1.27E-02 | 178680976 | 178681099 | NC_000005.9 |
| cg15906761 | ADAMTS2 | 2.59E-02 | 2.44 | -4.40919 | 7.18E-02 | 178623035 | 178623158 | NC_000005.9 |
| cg15726814 | ADAMTS2 | 8.51E-03 | -2.97 | -3.35415 | -3.45E-02 | 178594990 | 178595113 | NC_000005.9 |
| cg14830846 | ADAMTS2 | 1.95E-02 | -2.57 | -4.14085 | -2.37E-02 | 178763293 | 178763416 | NC_000005.9 |
| cg13909534 | ADAMTS2 | 4.42E-02 | 2.17 | -4.90092 | 1.48E-02 | 178772372 | 178772495 | NC_000005.9 |
| cg13380103 | ADAMTS2 | 4.88E-07 | -7.75 | 6.424432 | -1.02E-01 | 178770629 | 178770752 | NC_000005.9 |
| cg13041355 | ADAMTS2 | 1.15E-02 | -2.82 | -3.64612 | -2.66E-02 | 178622080 | 178622203 | NC_000005.9 |
| cg11791960 | ADAMTS2 | 9.81E-06 | 6.15 | 3.386856 | 9.36E-02 | 178667819 | 178667942 | NC_000005.9 |
| cg11200794 | ADAMTS2 | 3.34E-02 | -2.31 | -4.64329 | -1.15E-01 | 178684000 | 178684123 | NC_000005.9 |
| cg10997906 | ADAMTS2 | 1.37E-02 | 2.74 | -3.80863 | 1.23E-01 | 178753803 | 178753926 | NC_000005.9 |
| cg10213542 | ADAMTS2 | 1.16E-03 | 3.88 | -1.40923 | 1.50E-01 | 178692728 | 178692851 | NC_000005.9 |
| cg09540085 | ADAMTS2 | 2.05E-02 | -2.55 | -4.1917 | -2.54E-02 | 178650512 | 178650635 | NC_000005.9 |
| cg08999896 | ADAMTS2 | 2.00E-02 | -2.56 | -4.16721 | -6.87E-02 | 178685787 | 178685910 | NC_000005.9 |
| cg08902683 | ADAMTS2 | 6.39E-04 | -4.15 | -0.81434 | -4.37E-02 | 178748193 | 178748316 | NC_000005.9 |
| cg06495631 | ADAMTS2 | 3.31E-03 | 3.4 | -2.4382 | 1.47E-01 | 178692806 | 178692929 | NC_000005.9 |
| cg05656566 | ADAMTS2 | 2.55E-03 | 3.52 | -2.1828 | 7.60E-02 | 178593924 | 178594047 | NC_000005.9 |
| cg04184432 | ADAMTS2 | 1.59E-03 | -3.74 | -1.71635 | -6.09E-02 | 178622120 | 178622243 | NC_000005.9 |
| cg03259333 | ADAMTS2 | 3.16E-03 | -3.42 | -2.39088 | -1.33E-01 | 178592153 | 178592276 | NC_000005.9 |
| cg02946518 | ADAMTS2 | 1.44E-02 | -2.72 | -3.8586 | -1.24E-01 | 178683834 | 178683957 | NC_000005.9 |
| cg02497046 | ADAMTS2 | 2.24E-04 | -4.64 | 0.235545 | -1.13E-01 | 178636918 | 178637041 | NC_000005.9 |
| cg01791000 | ADAMTS2 | 1.66E-03 | -3.72 | -1.76193 | -3.27E-02 | 178687780 | 178687903 | NC_000005.9 |
| cg01231141 | ADAMTS2 | 7.07E-03 | 3.05 | -3.1753 | 1.50E-01 | 178692691 | 178692814 | NC_000005.9 |
| cg00799121 | ADAMTS2 | 4.04E-02 | 2.22 | -4.81816 | 1.27E-01 | 178741283 | 178741406 | NC_000005.9 |
| cg00562312 | ADAMTS2 | 4.49E-02 | 2.16 | -4.91405 | 3.04E-02 | 178752999 | 178753122 | NC_000005.9 |
| cg00096810 | ADAMTS2 | 4.55E-03 | 3.26 | -2.74795 | 9.91E-02 | 178753432 | 178753555 | NC_000005.9 |
| cg24643381 | ADAMTS20 | 3.10E-06 | -6.74 | 4.553948 | -8.64E-02 | 43941378 | 43941501 | NC_000012.11 |
| cg24606791 | ADAMTS20 | 6.54E-03 | 3.09 | -3.09988 | 1.35E-01 | 43946493 | 43946616 | NC_000012.11 |
| cg24116870 | ADAMTS20 | 5.43E-03 | 3.17 | -2.91956 | 1.06E-01 | 43946284 | 43946407 | NC_000012.11 |
| cg21911301 | ADAMTS20 | 1.96E-02 | 2.57 | -4.14899 | 4.41E-02 | 43946518 | 43946641 | NC_000012.11 |
| cg19619405 | ADAMTS20 | 2.71E-02 | 2.41 | -4.45157 | 6.98E-02 | 43945892 | 43946015 | NC_000012.11 |
| cg17459298 | ADAMTS20 | 2.84E-03 | 3.47 | -2.28949 | 4.78E-02 | 43946341 | 43946464 | NC_000012.11 |
| cg11751806 | ADAMTS20 | 9.88E-03 | 2.9 | -3.4969 | 5.54E-02 | 43945680 | 43945803 | NC_000012.11 |
| cg02925039 | ADAMTS20 | 1.57E-02 | 2.68 | -3.93924 | 6.93E-02 | 43945213 | 43945336 | NC_000012.11 |
| cg01345315 | ADAMTS20 | 3.72E-03 | 3.35 | -2.55286 | 9.78E-02 | 43945998 | 43946121 | NC_000012.11 |
| cg14349862 | ADAMTS3 | 1.40E-04 | 4.86 | 0.708603 | 6.05E-02 | 73178545 | 73178668 | NC_000004.11 |
| cg05797623 | ADAMTS3 | 8.53E-04 | 4.02 | -1.10074 | 2.16E-01 | 73178007 | 73178130 | NC_000004.11 |
| cg04812351 | ADAMTS3 | 5.77E-04 | 4.2 | -0.71264 | 2.13E-01 | 73177995 | 73178118 | NC_000004.11 |
| cg01664241 | ADAMTS3 | 5.38E-03 | 3.18 | -2.91121 | 7.32E-02 | 73435425 | 73435548 | NC_000004.11 |
| cg24329783 | ADAMTS4 | 1.56E-02 | 2.68 | -3.93469 | 4.89E-02 | 161160887 | 161161010 | NC_000001.10 |
| cg07354440 | ADAMTS4 | 1.69E-04 | 4.77 | 0.515912 | 2.47E-01 | 161168889 | 161169012 | NC_000001.10 |
| cg20156545 | ADAMTS5 | 4.25E-06 | 6.57 | 4.233858 | 2.58E-01 | 28335020 | 28335143 | NC_000021.8 |
| cg15237494 | ADAMTS5 | 6.09E-03 | 3.12 | -3.03084 | 1.02E-01 | 28339334 | 28339457 | NC_000021.8 |
| cg14740657 | ADAMTS5 | 4.22E-02 | 2.19 | -4.85925 | 6.57E-02 | 28337594 | 28337717 | NC_000021.8 |
| cg13601496 | ADAMTS5 | 4.41E-02 | 2.17 | -4.8973 | 3.21E-02 | 28339487 | 28339610 | NC_000021.8 |
| cg07771160 | ADAMTS5 | 3.76E-06 | 6.64 | 4.356961 | 1.79E-01 | 28335945 | 28336068 | NC_000021.8 |
| cg03294207 | ADAMTS5 | 1.12E-03 | 3.9 | -1.36917 | 1.23E-01 | 28336897 | 28337020 | NC_000021.8 |
| cg03202077 | ADAMTS5 | 8.46E-04 | 4.02 | -1.09316 | 7.66E-02 | 28338836 | 28338959 | NC_000021.8 |
| cg02800810 | ADAMTS5 | 1.88E-02 | 2.59 | -4.10788 | 7.27E-02 | 28340139 | 28340262 | NC_000021.8 |
| cg00646084 | ADAMTS5 | 8.41E-03 | 2.97 | -3.34255 | 9.57E-02 | 28337168 | 28337291 | NC_000021.8 |
| cg25229198 | ADAMTS6 | 1.15E-03 | 3.89 | -1.39498 | 1.21E-01 | 64660684 | 64660807 | NC_000005.9 |
| cg21033632 | ADAMTS6 | 2.39E-04 | -4.61 | 0.170024 | -1.41E-01 | 64486421 | 64486544 | NC_000005.9 |
| cg16580073 | ADAMTS6 | 1.16E-04 | 4.94 | 0.890789 | 1.10E-01 | 64446603 | 64446726 | NC_000005.9 |
| cg00299603 | ADAMTS6 | 1.22E-03 | -3.86 | -1.45411 | -3.15E-02 | 64494334 | 64494457 | NC_000005.9 |
| cg25490185 | ADAMTS7 | 9.87E-03 | -2.9 | -3.49563 | -2.89E-02 | 79094603 | 79094726 | NC_000015.9 |
| cg23506979 | ADAMTS7 | 4.09E-03 | 3.31 | -2.64339 | 1.96E-01 | 79090958 | 79091081 | NC_000015.9 |
| cg22183295 | ADAMTS7 | 4.34E-02 | -2.18 | -4.88308 | -9.65E-03 | 79099845 | 79099968 | NC_000015.9 |
| cg06752398 | ADAMTS7 | 2.06E-02 | -2.55 | -4.19227 | -9.62E-02 | 79053858 | 79053981 | NC_000015.9 |
| cg03762349 | ADAMTS7 | 1.17E-05 | 6.06 | 3.209496 | 9.44E-02 | 79060523 | 79060646 | NC_000015.9 |
| cg23886747 | ADAMTS8 | 1.85E-04 | 4.72 | 0.424834 | 1.64E-01 | 130297197 | 130297320 | NC_000011.9 |
| cg16453560 | ADAMTS8 | 8.65E-03 | 2.96 | -3.36968 | 2.57E-02 | 130298756 | 130298879 | NC_000011.9 |
| cg13464448 | ADAMTS8 | 5.81E-03 | 3.14 | -2.98603 | 3.30E-02 | 130297513 | 130297636 | NC_000011.9 |
| cg12270485 | ADAMTS8 | 1.74E-02 | -2.63 | -4.03695 | -8.90E-02 | 130299947 | 130300070 | NC_000011.9 |
| cg10521851 | ADAMTS8 | 6.42E-03 | 3.1 | -3.08227 | 6.25E-02 | 130297771 | 130297894 | NC_000011.9 |
| cg09134593 | ADAMTS8 | 7.54E-04 | 4.08 | -0.97782 | 6.98E-02 | 130297528 | 130297651 | NC_000011.9 |
| cg08182171 | ADAMTS8 | 6.86E-04 | -4.12 | -0.88401 | -9.34E-02 | 130291156 | 130291279 | NC_000011.9 |
| cg06944693 | ADAMTS8 | 4.20E-02 | -2.19 | -4.85468 | -2.81E-02 | 130299298 | 130299421 | NC_000011.9 |
| cg04268950 | ADAMTS8 | 7.96E-05 | 5.12 | 1.273129 | 1.27E-01 | 130298854 | 130298977 | NC_000011.9 |
| cg02518691 | ADAMTS8 | 1.18E-04 | 4.93 | 0.873593 | 1.67E-01 | 130297149 | 130297272 | NC_000011.9 |
| cg01033938 | ADAMTS8 | 6.44E-03 | 3.1 | -3.0844 | 7.69E-02 | 130298880 | 130299003 | NC_000011.9 |
| cg27203924 | ADAMTS9 | 3.65E-02 | -2.27 | -4.72496 | -3.02E-02 | 64627542 | 64627665 | NC_000003.11 |
| cg26061177 | ADAMTS9 | 1.73E-03 | 3.7 | -1.80224 | 6.24E-02 | 64670562 | 64670685 | NC_000003.11 |
| cg25859972 | ADAMTS9 | 3.05E-04 | 4.49 | -0.07502 | 1.29E-01 | 64670515 | 64670638 | NC_000003.11 |
| cg25581822 | ADAMTS9 | 3.61E-06 | 6.66 | 4.400094 | 1.47E-01 | 64534657 | 64534780 | NC_000003.11 |
| cg25157607 | ADAMTS9 | 2.79E-02 | 2.4 | -4.47671 | 1.31E-02 | 64672708 | 64672831 | NC_000003.11 |
| cg23070026 | ADAMTS9 | 4.81E-04 | 4.28 | -0.5305 | 2.47E-02 | 64672542 | 64672665 | NC_000003.11 |
| cg22177868 | ADAMTS9 | 7.23E-07 | 7.53 | 6.025938 | 1.39E-01 | 64667730 | 64667853 | NC_000003.11 |
| cg22118297 | ADAMTS9 | 9.75E-09 | 1.02E+01 | 10.36397 | 2.78E-01 | 64547310 | 64547433 | NC_000003.11 |
| cg22081832 | ADAMTS9 | 5.88E-04 | 4.19 | -0.73075 | 1.48E-01 | 64670459 | 64670582 | NC_000003.11 |
| cg21938436 | ADAMTS9 | 5.34E-03 | 3.18 | -2.90337 | 1.07E-01 | 64670985 | 64671108 | NC_000003.11 |
| cg21878275 | ADAMTS9 | 2.59E-02 | 2.44 | -4.40927 | 3.99E-02 | 64673501 | 64673624 | NC_000003.11 |
| cg21527616 | ADAMTS9 | 4.41E-10 | 1.24E+01 | 13.43783 | 2.91E-01 | 64547373 | 64547496 | NC_000003.11 |
| cg20180538 | ADAMTS9 | 1.63E-02 | -2.66 | -3.97162 | -3.97E-02 | 64559102 | 64559225 | NC_000003.11 |
| cg14187266 | ADAMTS9 | 7.31E-10 | 1.20E+01 | 12.93932 | 3.31E-01 | 64547346 | 64547469 | NC_000003.11 |
| cg12478384 | ADAMTS9 | 5.12E-03 | 3.26 | -2.7651 | 1.13E-01 | 64671764 | 64671887 | NC_000003.11 |
| cg12187213 | ADAMTS9 | 3.60E-02 | -2.27 | -4.71271 | -2.10E-02 | 64673741 | 64673864 | NC_000003.11 |
| cg11427510 | ADAMTS9 | 3.56E-02 | 2.28 | -4.7032 | 3.59E-02 | 64673495 | 64673618 | NC_000003.11 |
| cg10447977 | ADAMTS9 | 6.25E-06 | 6.37 | 3.842955 | 2.42E-01 | 64598405 | 64598528 | NC_000003.11 |
| cg07891473 | ADAMTS9 | 5.28E-03 | 3.19 | -2.89182 | 6.86E-02 | 64670640 | 64670763 | NC_000003.11 |
| cg07777540 | ADAMTS9 | 3.70E-03 | 3.35 | -2.54728 | 2.16E-02 | 64524911 | 64525034 | NC_000003.11 |
| cg05501868 | ADAMTS9 | 1.01E-02 | 2.88 | -3.52036 | 1.02E-01 | 64516087 | 64516210 | NC_000003.11 |
| cg04421973 | ADAMTS9 | 2.77E-02 | 2.4 | -4.47216 | 4.20E-02 | 64670907 | 64671030 | NC_000003.11 |
| cg04366011 | ADAMTS9 | 9.40E-03 | 2.97 | -3.35728 | 4.95E-02 | 64671477 | 64671600 | NC_000003.11 |
| cg03427905 | ADAMTS9 | 4.25E-03 | 3.29 | -2.68162 | 7.47E-02 | 64671378 | 64671501 | NC_000003.11 |
| cg02810826 | ADAMTS9 | 5.75E-03 | -3.15 | -2.97518 | -6.20E-02 | 64504415 | 64504538 | NC_000003.11 |
| cg02246130 | ADAMTS9 | 1.44E-09 | 1.15E+01 | 12.26737 | 3.15E-01 | 64547152 | 64547275 | NC_000003.11 |
| cg01635742 | ADAMTS9 | 1.40E-05 | -5.97 | 3.030119 | -7.95E-02 | 64561075 | 64561198 | NC_000003.11 |
| cg01424460 | ADAMTS9 | 2.93E-02 | 2.38 | -4.52139 | 2.46E-02 | 64673301 | 64673424 | NC_000003.11 |
| cg00344411 | ADAMTS9 | 8.61E-11 | 1.38E+01 | 15.0353 | 3.98E-01 | 64547108 | 64547231 | NC_000003.11 |
| ch.9.357218F | ADAMTSL1 | 8.55E-04 | 4.07 | -1.04933 | 4.72E-02 | 18618131 | 18618254 | NC_000009.11 |
| cg14502847 | ADAMTSL1 | 2.85E-04 | -4.53 | -0.00656 | -1.41E-01 | 18490009 | 18490132 | NC_000009.11 |
| cg14174232 | ADAMTSL1 | 9.22E-03 | 2.93 | -3.43016 | 2.61E-02 | 18909891 | 18910014 | NC_000009.11 |
| cg14003978 | ADAMTSL1 | 3.16E-04 | 4.48 | -0.11223 | 2.06E-01 | 18862115 | 18862238 | NC_000009.11 |
| cg13468759 | ADAMTSL1 | 1.28E-07 | 8.53 | 7.7743 | 5.61E-02 | 18605667 | 18605790 | NC_000009.11 |
| cg00116234 | ADAMTSL1 | 4.85E-03 | 3.23 | -2.81038 | 3.65E-02 | 18474243 | 18474366 | NC_000009.11 |
| cg21202204 | ADAMTSL2 | 3.53E-06 | -6.67 | 4.421045 | -1.98E-01 | 136398165 | 136398288 | NC_000009.11 |
| cg18783801 | ADAMTSL2 | 9.14E-03 | 2.93 | -3.42237 | 3.39E-02 | 136419751 | 136419874 | NC_000009.11 |
| cg14389669 | ADAMTSL2 | 2.32E-04 | 4.62 | 0.200144 | 9.75E-02 | 136399386 | 136399509 | NC_000009.11 |
| cg14201417 | ADAMTSL2 | 1.16E-02 | 2.82 | -3.65046 | 4.01E-02 | 136400057 | 136400180 | NC_000009.11 |
| cg13872238 | ADAMTSL2 | 1.27E-06 | 7.22 | 5.453845 | 1.78E-01 | 136430793 | 136430916 | NC_000009.11 |
| cg13748845 | ADAMTSL2 | 7.83E-03 | -3 | -3.27359 | -4.04E-02 | 136434290 | 136434413 | NC_000009.11 |
| cg13464321 | ADAMTSL2 | 7.42E-03 | -3.03 | -3.22101 | -9.92E-02 | 136422991 | 136423114 | NC_000009.11 |
| cg13459291 | ADAMTSL2 | 1.14E-02 | -2.83 | -3.63524 | -4.31E-02 | 136433277 | 136433400 | NC_000009.11 |
| cg11896113 | ADAMTSL2 | 3.05E-02 | -2.36 | -4.55944 | -1.67E-02 | 136397740 | 136397863 | NC_000009.11 |
| cg00742898 | ADAMTSL2 | 2.80E-02 | -2.4 | -4.48117 | -2.32E-02 | 136431882 | 136432005 | NC_000009.11 |
| cg18734428 | ADAMTSL3 | 9.23E-03 | 2.93 | -3.43198 | 5.92E-02 | 84322277 | 84322400 | NC_000015.9 |
| cg15324185 | ADAMTSL3 | 1.08E-02 | -2.85 | -3.5815 | -2.49E-02 | 84612808 | 84612931 | NC_000015.9 |
| cg14598478 | ADAMTSL3 | 1.24E-05 | -6.03 | 3.148488 | -1.95E-01 | 84363061 | 84363184 | NC_000015.9 |
| cg14230666 | ADAMTSL3 | 1.88E-02 | 2.59 | -4.10758 | 8.89E-02 | 84322946 | 84323069 | NC_000015.9 |
| cg11611600 | ADAMTSL3 | 1.56E-02 | 2.68 | -3.93073 | 6.44E-02 | 84323154 | 84323277 | NC_000015.9 |
| cg08115297 | ADAMTSL3 | 2.90E-02 | -2.38 | -4.51256 | -2.81E-02 | 84706833 | 84706956 | NC_000015.9 |
| cg05315510 | ADAMTSL3 | 6.07E-04 | -4.18 | -0.76203 | -3.71E-02 | 84491709 | 84491832 | NC_000015.9 |
| cg04138185 | ADAMTSL3 | 1.12E-02 | 2.84 | -3.61308 | 7.37E-02 | 84322584 | 84322707 | NC_000015.9 |
| cg01152302 | ADAMTSL3 | 1.16E-04 | 4.94 | 0.890668 | 1.08E-01 | 84323770 | 84323893 | NC_000015.9 |
| cg27630771 | ADAMTSL4 | 6.62E-04 | 4.14 | -0.8494 | 8.61E-02 | 150533123 | 150533246 | NC_000001.10 |
| cg26875450 | ADAMTSL4 | 2.55E-02 | 2.44 | -4.39265 | 2.95E-02 | 150530531 | 150530654 | NC_000001.10 |
| cg24441324 | ADAMTSL4 | 1.33E-04 | 4.88 | 0.758366 | 2.73E-01 | 150523231 | 150523354 | NC_000001.10 |
| cg21996039 | ADAMTSL4 | 2.08E-03 | -3.61 | -1.98132 | -3.19E-02 | 150525424 | 150525547 | NC_000001.10 |
| cg15107203 | ADAMTSL4 | 1.12E-05 | 6.08 | 3.251178 | 1.74E-01 | 150529170 | 150529293 | NC_000001.10 |
| cg14037218 | ADAMTSL4 | 2.12E-04 | 4.66 | 0.287655 | 1.83E-01 | 150522367 | 150522490 | NC_000001.10 |
| cg12974394 | ADAMTSL4 | 1.54E-04 | 4.81 | 0.607855 | 1.82E-01 | 150522266 | 150522389 | NC_000001.10 |
| cg09622957 | ADAMTSL4 | 1.97E-02 | -2.57 | -4.1511 | -1.54E-02 | 150521190 | 150521313 | NC_000001.10 |
| cg06185850 | ADAMTSL4 | 2.49E-02 | 2.46 | -4.37028 | 2.45E-02 | 150529828 | 150529951 | NC_000001.10 |
| cg01922433 | ADAMTSL4 | 2.21E-04 | 4.64 | 0.24627 | 6.39E-02 | 150529761 | 150529884 | NC_000001.10 |
| cg24892628 | ADAMTSL5 | 1.44E-03 | 3.78 | -1.61653 | 1.52E-01 | 1509841 | 1509964 | NC_000019.9 |
| cg22994198 | ADAMTSL5 | 1.54E-03 | 3.75 | -1.68878 | 7.54E-02 | 1508528 | 1508651 | NC_000019.9 |
| cg17720286 | ADAMTSL5 | 4.71E-05 | -5.37 | 1.801427 | -8.49E-02 | 1513941 | 1514064 | NC_000019.9 |
| cg17147471 | ADAMTSL5 | 3.98E-04 | 4.37 | -0.34257 | 9.43E-02 | 1510584 | 1510707 | NC_000019.9 |
| cg15002904 | ADAMTSL5 | 1.47E-02 | 2.71 | -3.87562 | 8.99E-02 | 1510692 | 1510815 | NC_000019.9 |
| cg13434396 | ADAMTSL5 | 4.44E-05 | 5.4 | 1.862068 | 1.19E-01 | 1508555 | 1508678 | NC_000019.9 |
| cg09791746 | ADAMTSL5 | 3.84E-05 | 5.47 | 2.007186 | 2.31E-01 | 1510494 | 1510617 | NC_000019.9 |
| cg00658405 | ADAMTSL5 | 1.15E-02 | 2.83 | -3.63913 | 9.33E-02 | 1510500 | 1510623 | NC_000019.9 |
| cg17904988 | ADAMTS4 | 5.96E-05 | 5.26 | 1.563915 | 1.79E-01 | 161168451 | 161168574 | NC_000001.10 |
| cg17166812 | ADAMTS4 | 1.64E-02 | 2.66 | -3.98213 | 7.49E-02 | 161169574 | 161169697 | NC_000001.10 |
| cg14448116 | ADAMTS4 | 6.01E-04 | 4.18 | -0.75186 | 8.91E-02 | 161170110 | 161170233 | NC_000001.10 |
| cg14382215 | ADAMTS4 | 2.53E-04 | 4.58 | 0.113222 | 2.14E-01 | 161169007 | 161169130 | NC_000001.10 |
| cg11801851 | ADAMTS4 | 2.88E-05 | 5.61 | 2.299423 | 2.29E-01 | 161169164 | 161169287 | NC_000001.10 |
| cg10493436 | ADAMTS4 | 1.04E-04 | 5 | 1.005824 | 2.64E-01 | 161168957 | 161169080 | NC_000001.10 |
| cg08545169 | ADAMTS4 | 2.60E-05 | 5.66 | 2.399776 | 1.65E-01 | 161169143 | 161169266 | NC_000001.10 |
| cg06760507 | ADAMTS4 | 1.09E-03 | 3.91 | -1.34241 | 1.93E-01 | 161168145 | 161168268 | NC_000001.10 |
| cg04436964 | ADAMTS4 | 4.84E-02 | 2.12 | -4.98285 | 5.36E-02 | 161167745 | 161167868 | NC_000001.10 |
| cg04263215 | ADAMTS4 | 1.48E-04 | 4.83 | 0.64617 | 2.53E-01 | 161168856 | 161168979 | NC_000001.10 |
